# SANS contrast matching for the unambiguous localization of anionic dye in cationic surfactant micelles†

**DOI:** 10.1039/d3na00556a

**Published:** 2023-09-11

**Authors:** Wenke Müller, Ralf Schweins, Bernd Nöcker, Hans Egold, Yvonne Hannappel, Klaus Huber

**Affiliations:** a Institut Laue-Langevin, DS/LSS 71 Avenue des Martyrs 38000 Grenoble France we-mue@gmx.net; b KAO Germany GmbH Pfungstädter Straße 98-100 64297 Darmstadt Germany; c Universität Paderborn Warburger Straße 100 33098 Paderborn Germany; d Universität Bielefeld Universitätsstrasse 25 33615 Bielefeld Germany

## Abstract

Contrast variation in small-angle neutron scattering (SANS) was successfully applied to localize the anionic azo dye Blue in co-assemblies with the cationic surfactant dodecyltrimethylammoniumbromide (DTAB). For this purpose, the scattering contrast between DTAB and the aqueous solvent was eliminated by SANS contrast matching, leaving only the scattering signal from Blue to be detected. Results obtained by contrast matching were confirmed by NOESY NMR-spectroscopy, showing that Blue interacts with the positively charged DTAB head groups and with up to the 4^th^ neighbouring methylene group of the DTAB C_12_-alkyl chain. Its localization in the outer layer of the Blue–DTAB co-assembly explains the uniaxial growth of spheroidal DTAB micelles to wormlike micelles with increasing [Blue] : [DTAB] ratio from 0 : 1 to 1 : 3. This is in line with the concept of the packing parameter for amphiphilic substances.

## Abstract 2

The complementary use of SANS contrast matching and NMR-spectroscopy permits the unambiguous localization of an anionic dye in dodecyl trimethyl-ammonium bromide micelles.

## Graphical abstract

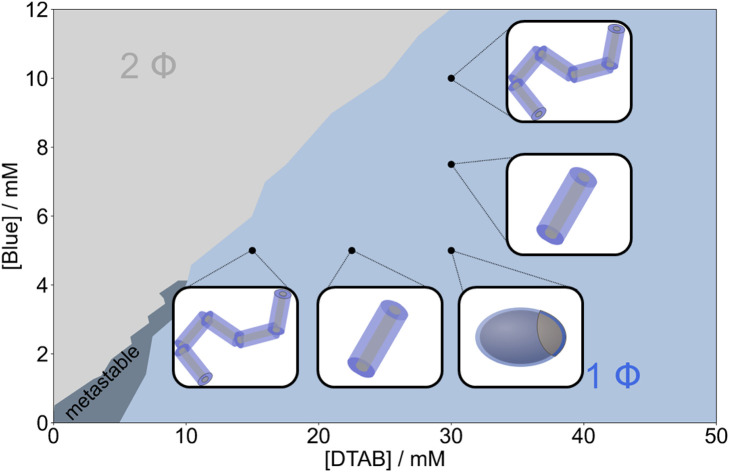

## Introduction

Systems capable of interacting with and responding to their environment are interesting for the design of sensor applications or molecular switches.^1^ For instance, systems of wormlike micelles (WLMs) were found to respond to various triggers, by forming cross-linked networks of these micelles. Such a response results in a viscosity increase of the solution as directly observable parameter.^2–4^ Depending on the system, responsivity to composition, pH, ionic strength, temperature, redox reactions, light and even CO_2_ may be achieved.^2,5–11^ WLMs are frequently formed by small amphiphilic surfactant molecules upon concentration increase or addition of inorganic salt, another surfactant or hydrotropic solutes.^4,9,10,12–14^ This provides a versatile, simple and huge toolbox for stimuli-responsive systems.^2^ Given the variety of possible components of WLM-forming systems, it is important to fundamentally understand intermolecular interactions leading to observable responses. Responsivity to pH, redox conditions or irradiation is frequently achieved by the interaction of surfactant micelles with a compound sensitive to corresponding changes.^2,7,15,16^ In order to understand the mechanism of responsivity to permit systematic improvement or rational design of similar systems, the localization of such a compound within the surfactant micelle needs to be known.

It is possible to obtain information on the localization of a compound in a surfactant micelle using nuclear magnetic resonance (NMR)-spectroscopy. Use of the 2-dimensional Nuclear Overhauser Effect spectroscopy (NOESY) is particularly helpful in this regard, as it provides information on spatial proximity.^17,18^ In some cases, however, an unambiguous localization of a solute in a co-assembly is not possible with NOESY due to overlap of resonances or ambiguity in peak assignment.^5,19^ Furthermore, NOESY does not provide information on the morphology of the co-assemblies.

Small-angle neutron scattering (SANS) permits the elucidation of assembly morphology while also providing the possibility to locate an added compound within that assembly using contrast variation. This technique can either be used in combination with NOESY to confirm and extend findings, but may also be applied as stand-alone-method.

SANS contrast variation was previously employed to explain morphological transitions of mixed surfactant micelles upon variation of the ratio between both surfactants by identifying their mutual arrangement in the mixed micellar assembly.^20,21^ It was furthermore successfully used to locate the protein hydrophobin in the outer shell of its co-assemblies with either ionic or non-ionic surfactant and to identify its folding state.^22^ Penfold *et al.* located small organic fragrance molecules in micelles formed by the non-ionic surfactant dodecaethylene glycol monododecyl ether (C_12_E_12_) by recording SANS curves under different contrast conditions.^23^ However, Penfold *et al.* did not perform contrast matching, resulting in ambiguity concerning the solubilisation locus of phenyl ethanol.

Herein, the value and feasibility of SANS contrast matching for the localization of an organic azo dye in micelles of a low molecular weight surfactant is demonstrated. The SANS experiments are complemented by NMR experiments and the results jointly discussed.

**Fig. 1:**
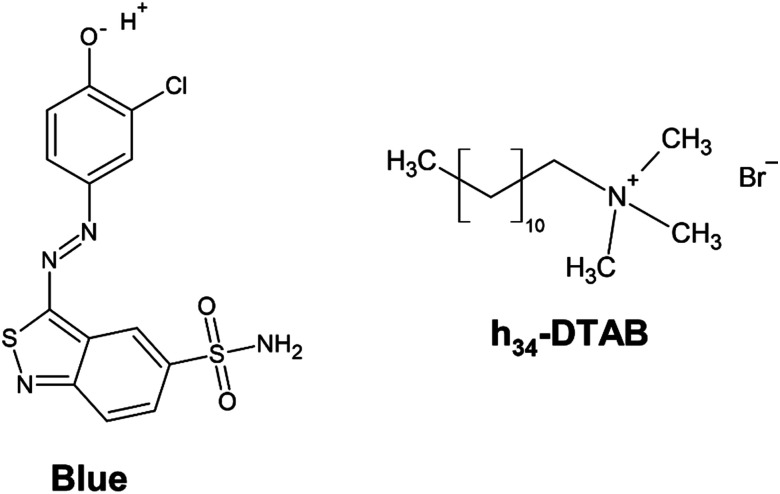
Chemical structure of 3-(3-chloro-4-hydroxy-phenylazo)benzo[*c*]isothiazole-5-sulfonamide (Blue) and of completely hydrogenated dodecyltrimethylammoniumbromide (h_34_-DTAB). In the aqueous, alkaline NaHCO_3_/Na_2_CO_3_ buffer system (pH = 10.5 or pD = 10.7, *I* ≈ 0.25 M) used as a solvent in this work, the phenolic hydroxyl group of Blue is deprotonated.

WLM formation of the cationic surfactant dodecyltrimethylammoniumbromide (DTAB) and the anionic azo dye Blue (Fig. 1) was studied as a model system. The interaction between oppositely charged azo dyes and surfactants has received attention as their manifold intermolecular interactions render them as promising building blocks in colloidal chemistry.^14,24,25^ Apart from hydrophobic and electrostatic interactions, π–π stacking of the dye was suggested to have a template-effect on assembly morphology.^26^ The co-assembly of Blue and DTAB was studied in an aqueous NaHCO_3_/Na_2_CO_3_ buffer with pH = 10.5 and ionic strength *I* ≈ 0.25 M.

## Experimental

### Chemicals and sample preparation

Blue (HC Blue 18, ≥99.8%) was provided by KAO GmbH, Germany. Hydrogenated dodecyltrimethylammoniumbromide (h_34_-DTAB, 99%) was obtained from abcr GmbH, Germany. Completely deuterated dodecyltrimethylammoniumbromide (d_34_-DTAB, 99% isotopic purity) was obtained from INNOVACHEM SAS, France. Tail-deuterated dodecyltrimethylammoniumbromide (d_25_-DTAB, 99.1% isotopic purity) was obtained from C/D/N Isotopes Inc., Canada. The buffer salts sodium carbonate Na_2_CO_3_ (≥99.8%) and sodium bicarbonate NaHCO_3_ (≥99.7%) were obtained from Sigma Aldrich Chemie GmbH, Germany. MilliQ water was used to prepare the NaHCO_3_/Na_2_CO_3_ buffer solutions (pH = 10.5, ionic strength *I* ≈ 0.25 M) for samples for viscosity measurements, Cryo-TEM measurements and determination of the phase diagram by visual inspection. D_2_O was used to prepare the NaHCO_3_/Na_2_CO_3_ buffer solutions (pD = 10.7, ionic strength *I* ≈ 0.25 M) for SANS and NMR samples. A mixture of 50 vol% H_2_O and 50 vol% D_2_O was used to prepare the NaHCO_3_/Na_2_CO_3_ buffer solutions (pH = 10.5, ionic strength *I* ≈ 0.25 M) for the SANS sample containing Blue at a concentration of [Blue] = 5 mM and h_34_-DTAB at a concentration of [h_34_-DTAB] = 30 mM. In all cases, the buffer contained NaHCO_3_ at a concentration of 0.021 M and Na_2_CO_3_ at a concentration of 0.079 M, which resulted in the given ionic strength (*I* ≈ 0.25 M) and pH = 10.5 (pD = 10.7). D_2_O (99.90% D) was obtained from Eurisotop, France or Sigma Aldrich Chemie GmbH, Germany. Chemicals were used without further purification. Samples were prepared from stock solutions, followed by a minimum equilibration time of 20 h at room temperature prior to analysis.

### Phase diagram

The phase diagram was established by stepwise addition of a 15 mM stock solution of Blue to solutions containing h_34_-DTAB at different concentrations, depending on the concentration range to be probed. NaHCO_3_/Na_2_CO_3_ buffer solution (pH = 10.5, ionic strength *I* ≈ 0.25 M) was prepared with MilliQ water and used as a solvent in all cases. After each addition of dye stock solution, the sample was vortexed for approximately 30 s and its visual appearance recorded immediately. Some samples were kept and visually inspected after 24 h to investigate long-term stability. Phase diagrams were established at room temperature (≈22 °C).

### Cryo-TEM

The cryo-transmission electron microscopy (Cryo-TEM) measurements were performed on a JEOL JEM-2200FS electron microscope (JEOL, Freising, Germany) operating at 200 kV acceleration voltage and equipped with an “OneView” CMOS camera (Gatan, Pleasanton, CA, USA). 3 μL of a sample containing Blue at a concentration of 10 mM and DTAB at a concentration of 30 mM in an aqueous NaHCO_3_/Na_2_CO_3_ buffer (pH = 10.5, ionic strength *I* ≈ 0.25 M) were deposited on the surface of a lacey carbon film coated grid (200 Mesh, Cu, Science Services GmbH, Munich, Germany) and vitrified by a Leica blotting and plunging device operating at room temperature (Leica EM GP, Leica Mikrosysteme Vertrieb GmbH, Wetzlar, Germany). The samples were plunged into liquid ethane, which was cooled with liquid nitrogen to achieve sufficiently fast cooling and freezing without formation of ice crystals. Afterwards the sample was transferred into a cryo transfer and tomography holder (Fischione, Model 2550, E.A. Fischione Instruments, Pittsburgh, PA, USA). The images captured were afterwards processed with a digital imaging processing program (Digital Micrograph®, Version 3.21, GMS 3, Gatan, Pleasanton, CA, USA).

### Viscosity

The kinematic viscosities *ν* of solutions containing Blue and h_34_-DTAB in an aqueous NaHCO_3_/Na_2_CO_3_ buffer (pH = 10.5, ionic strength *I* ≈ 0.25 M) were determined using an Ubbelohde viscometer (Type 52710/I Schott, viscometer constant *K* = 0.00933 mm^2^ s^−2^). Viscosity measurements were performed at 25 °C. The kinematic viscosity was obtained by multiplying the viscometer constant *K* by the time *t* the solution needed to drop from the first to the second mark of the Ubbelohde viscometer: *ν* = *K* × *t*.

### Small-angle neutron scattering

After their preparation from stock solutions, SANS samples were filtered (MACHEREY-NAGEL, CHROMAFIL Xtra H-PTFE syringe filters, pore size 0.2 μm) into dust-free sample vials and equilibrated for a minimum of 20 h at room temperature.

In a first step, contrast matching of a d_25_-DTAB/d_34_-DTAB mixture to the NaHCO_3_/Na_2_CO_3_ buffer prepared in 100% D_2_O was performed. For this purpose, four solutions containing a total DTAB concentration of 30 mM but varying proportions of d_25_-DTAB and d_34_-DTAB were prepared and their SANS-curves recorded. Square roots of resulting forward scattering intensities 
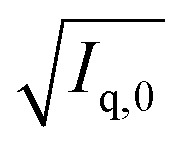
 were plotted against the volume fraction of d_25_-DTAB contained in the sample and the match composition of d_25_-DTAB : d_34_-DTAB determined to 46 : 54 (v/v) by linear extrapolation towards 
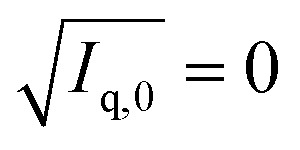
. This match composition was experimentally confirmed by recording the SANS curve of a corresponding solution, which is shown in the ESI (Fig. SI2†). “DTAB-matched” samples with eliminated scattering contrast between DTAB and the solvent were therefore always prepared with a 46 : 54 (v/v) mixture of d_25_-DTAB : d_34_-DTAB in a NaHCO_3_/Na_2_CO_3_ buffer in 100% D_2_O.

In a second step, “full contrast” SANS curves were recorded, where Blue and DTAB show non-zero contrast relative to the solvent. In the first set of experiments, full contrast SANS curves were obtained by dissolving the above-mentioned 46 : 54 mixture of d_25_-DTAB and d_34_-DTAB in a NaHCO_3_/Na_2_CO_3_ buffer with a mixture of 50 vol% H_2_O and 50 vol% D_2_O instead of 100 vol% D_2_O. This only concerns the sample containing Blue at a concentration of [Blue] = 5 mM and DTAB at a concentration of [DTAB] = 30 mM. In all other cases, full contrast was achieved by dissolving 100% h_34_-DTAB in a NaHCO_3_/Na_2_CO_3_ buffer with 100 vol% D_2_O. To permit comparison between full contrast SANS curves of the sample with [Blue] = 5 mM and [DTAB] = 30 mM to all other full contrast SANS curves, this SANS curve and corresponding fits were multiplied with a scaling factor *f* for display. The scaling factor is intended to account for scattering length density (SLD) differences and was estimated according to theoretical SLD differences to:

1 *f* = (SLD_h_34_-DTAB_ − SLD_100% D_2_O solvent_)^2^/(SLD_matched DTAB_ − SLD_50% D_2_O solvent_)^2^ = [(−0.224 − 6.376) × 10^−6^ Å^−2^]^2^/[(6.376 − 2.918) × 10^−6^ Å^−2^]^2^ ≈ 3.6

In eqn (1) SLD_h_34_-DTAB_ is the theoretical SLD of h_34_-DTAB and SLD_matched DTAB_ the SLD of the mixture of d_25_-DTAB and d_34_-DTAB (46 : 54). SLD_100% D_2_O solvent_ is the SLD of the solvent containing 100 vol% D_2_O. SLD_50% D_2_O solvent_ is the SLD of the solvent consisting of 50 vol% H_2_O and 50 vol% D_2_O. This scaling was solely done for plotting the full contrast SANS curve of the sample with [Blue] = 5 mM and [DTAB] = 30 mM for comparison to full contrast SANS curves of other samples obtained under different SLD conditions. Fitting was performed with the unscaled data.

In a third step, SANS curves of “DTAB-matched” samples were recorded, where the scattering contrast of DTAB relative to the solvent is zero but Blue shows a scattering contrast with the respective solvent. For this purpose, the above-described mixture of 46 : 54 (v/v) mixture of d_25_-DTAB : d_34_-DTAB in a NaHCO_3_/Na_2_CO_3_ buffer in 100% D_2_O was used.

SANS was performed at the small-angle neutron scattering instrument D11 at the Institut Laue-Langevin (Grenoble, France). Different setups were used: (1) identification of the match point and the measurement of the full contrast and the DTAB-matched sample containing [Blue] = 5 mM and [DTAB] = 30 mM were carried out at three sample-to-detector distances (28 m, collimation 28 m), (8 m, collimation 8 m), (1.7 m, collimation 4.0 m) at a neutron wavelength of 6 Å to cover a *q*-range of 0.002 Å^−1^ to 0.5 Å^−1^. A circular neutron beam with a diameter of 15 mm was used. (2) Full contrast and DTAB-matched samples containing [Blue] : [DTAB] at a ratio of 1 : 3 were measured at three sample-to-detector distances (38.0 m, collimation 40.5 m), (10.5 m, collimation 10.5 m), (1.7 m, collimation 2.5 m) at a neutron wavelength of 6 Å to cover a *q*-range of 0.0014 Å^−1^ to 0.5 Å^−1^. A circular neutron beam with a diameter of 14 mm was used. (3) Full contrast and DTAB-matched samples containing [Blue] : [DTAB] at a ratio of 1 : 4 or 1 : 4.5 were measured at three sample-to-detector distances (38.0 m, collimation 40.5 m), (10.5 m, collimation 10.5 m), (2.5 m, collimation 2.5 m) at a neutron wavelength of 6 Å to cover a *q*-range of 0.0014 Å^−1^ to 0.5 Å^−1^. A circular neutron beam with a diameter of 14 mm was used. Neutrons were detected with a ^3^He-detector (Reuter-Stokes multi-tube detector consisting of 256 tubes with a tube diameter of 8 mm and a pixel size of 8 mm × 4 mm), detector images azimuthally averaged, corrected to the transmission of the direct beam and scaled to absolute intensity using the Mantid software.^27,28^ Empty cell and solvent scattering were subtracted from the scattering curves.^29^ SANS data were collected at a sample temperature of 25 °C.

### NMR-spectroscopy

Samples for NMR-spectroscopy were prepared from stock solutions. A NaHCO_3_/Na_2_CO_3_ buffer (pD = 10.7, ionic strength *I* ≈ 0.25 M) prepared in D_2_O was used as a solvent in all cases. Sample solutions were filtered (MACHEREY-NAGEL, CHROMAFIL Xtra H-PTFE syringe filters, pore size 0.2 μm) into the NMR-tube. ^1^H-NMR and Nuclear Overhauser Effect spectroscopy (NOESY) spectra were recorded with a NMR Ascent 700 spectrometer (700 MHz) equipped with a cryogenic probe with z-gradient at 298 K. ^1^H-NMR chemical shifts were referenced to residual HDO.^30^

## Data analysis

The absolute scattering intensity *I*_q_ arising from particles with a volume *V*_part_ and a volume fraction of *ϕ* = *N* × *V*_part_/*V*_tot_ with *N* being the number of particles and *V*_tot_ the total sample volume is generally described by the following equation:^29^

2 *I*_q_ = *KϕV*_part_*P*(*q*)*S*(*q*) + *B*_incoh_

*K* is the contrast factor and *P*(*q*) is the particle form factor, which describes *q*-dependent modulations in scattered intensity caused by interferences between neutron waves scattered from the same particle. It is therefore determined by particle geometry. For small *q* it approaches *P*(*q* → 0) = 1.^29^*S*(*q*) denotes the structure factor which arises due to spatial ordering of different particles. In case of negligible inter-particular interactions and ordering, *e.g.* in sufficiently dilute solutions, *S*(*q*) tends to one.^29^*B*_incoh_ is a background signal arising due to incoherent scattering and is therefore termed “incoherent background”. Scattering contrast is caused by differences in SLD between the particle and the solvent. In case of a simple system, where the particles possess a constant SLD_1_ which is homogeneously distributed throughout the whole particle, and the solvent possesses a homogeneous SLD_solv_, *K* depends on their difference according to:^29^

3 *K* = (SLD_1_ − SLD_solv_)^2^

For SANS data presented in this work, the particle form factor used to describe an experimental SANS curve was chosen based on: (1) preliminary information on the system, (2) characteristic features of the SANS-curves, (3) fit quality and (4) morphological transitions expected from existing models and similar systems. The SasView small-angle scattering analysis software with implemented form- and structure factor models was used for analysis of experimental SANS curves in most cases.^31^ For generating and fitting the form factor model of a triaxial ellipsoidal shell, the SASfit software package was used.^32^ For form factor fits, the volume fraction of scattering particles was derived from the known molar concentration of scatterers and their partial molar volumes. For this purpose, it was assumed that all Blue- and DTAB-molecules, which are present in solution, participate in micelle formation. Partial molar volumes are displayed in the ESI (Table SI1†). SLD_1_, *B*_incoh_ and size parameters were fitted. For form factor fitting of core–shell structures two fit strategies were used: In a first approach, the SLD of the shell was kept constant. In a second approach it was fitted. Details regarding this procedure can be found in the discussion. The consideration of a structure factor was only necessary for fitting SANS curves from pure DTAB micelles in the absence of Blue. In all other cases no relevant inter-particle ordering was observed.

## Results and discussion

### Phase diagram and viscosity

**Fig. 2:**
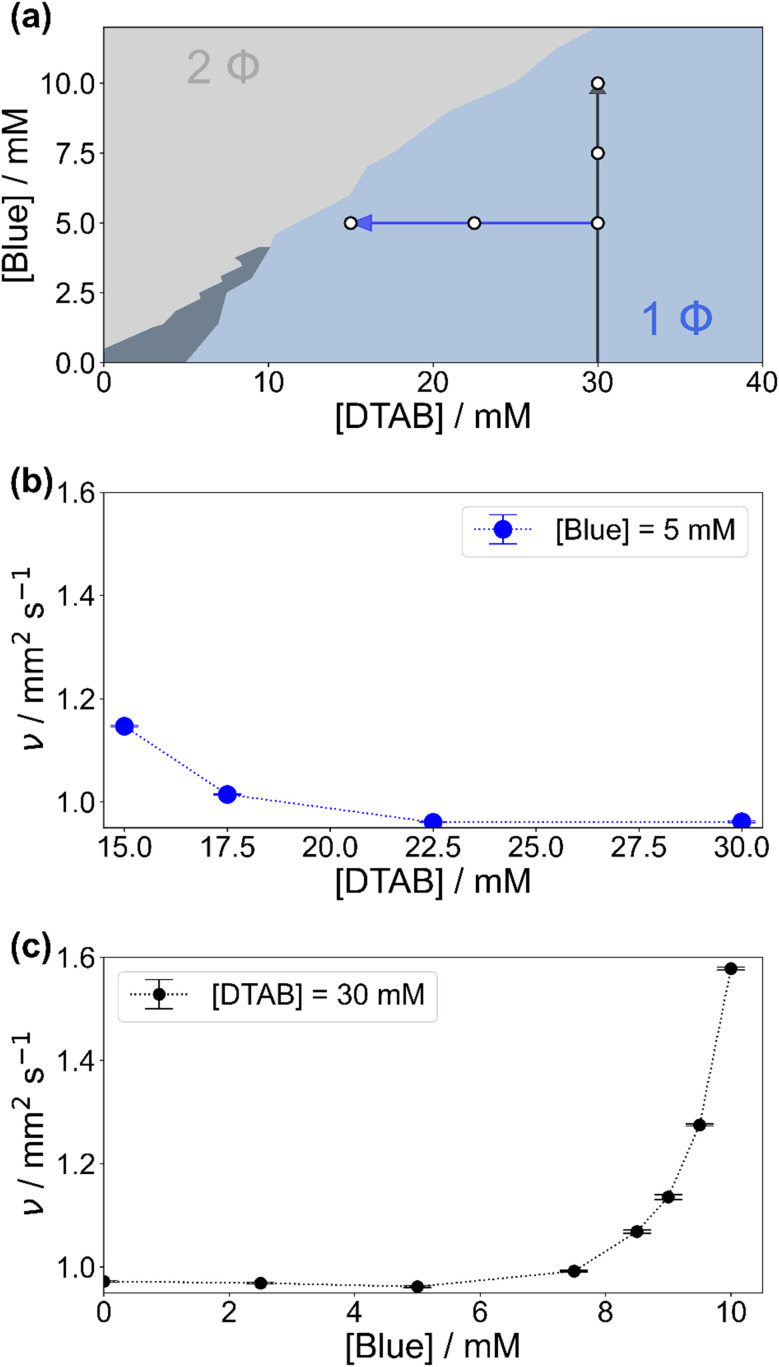
(a) Phase diagram of solutions containing Blue and DTAB in a NaHCO_3_/Na_2_CO_3_ buffer with pH = 10.5 and ionic strength *I* ≈ 0.25 M at room temperature. The light blue 1 Φ region covers stable solutions. The dark slate grey area denotes a metastable region within which samples are stable for 30 s after mixing but precipitate within 1 h to 24 h. In the 2 Φ region phase separation occurs due to the formation of solid Blue–DTAB complexes. Arrows indicate an increase of the kinematic viscosity of samples at 25 °C according to (b) and (c). White circles denote the composition of samples studied by SANS. (b) Kinematic viscosity of solutions containing [Blue] = 5 mM and varying concentrations of DTAB at 25 °C. (c) Kinematic viscosity of solutions containing [DTAB] = 30 mM and varying concentrations of Blue at 25 °C.

Fig. 2(a) shows the concentration-dependent phase diagram of solutions containing Blue and DTAB at room temperature. Phase separation of homogeneous solutions into a liquid and a solid phase occurs within 30 s after mixing once a certain Blue : DTAB ratio is exceeded.^33^ The solid phase was observed to be a crystalline precipitate corresponding to highly ordered Blue–DTAB complexes, which are likely caused by lamellar assembly of both components.^24,33^

To understand phenomena leading to phase separation, the viscosity of samples in the 1-phase region was investigated. Fig. 2(b) and (c) show the development of the kinematic viscosity *ν* of Blue–DTAB solutions at a constant concentration of Blue ([Blue]) as a function of DTAB concentration ([DTAB]) and at a constant concentration of DTAB as a function of [Blue] respectively. Both figures show that the kinematic viscosity of samples increases with increasing Blue : DTAB ratio, *i.e.* upon approaching the phase transition threshold. This increase is not linear but rises with proximity to the 2-phase region. Considering existing literature, similar trends were observed for the change in solution zero shear viscosity *η*_0_ upon addition of hydrotrope to surfactant solutions and are often related to morphological changes in the sample.^9,14^ The observation of a viscosity increase when approaching the phase transition threshold points towards the formation of WLMs, their entanglement and network formation.^3,9,34,35^

WLM formation from cationic surfactants is a frequently observed phenomenon and can be induced by various triggers.^4^ These include the addition of salt or hydrotropes such as phenol and phenolate derivatives or the addition of negatively charged azo dyes.^9,10,12,14^ For this reason, the presence of WLMs in solutions of the negatively charged azo dye Blue and the positively charged surfactant DTAB close to the phase transition threshold was strongly expected.

**Fig. 3:**
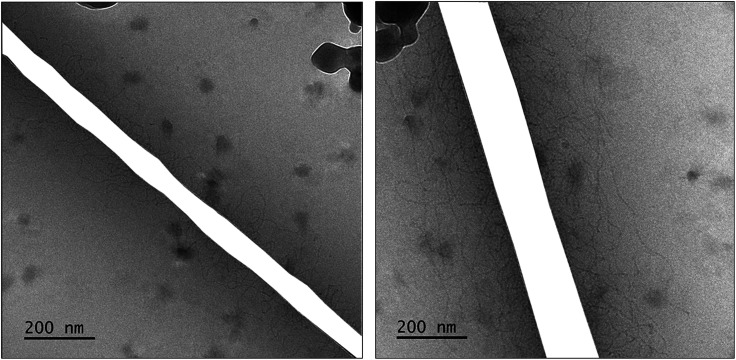
Cryo-TEM images of a solution containing [Blue] = 10 mM and [DTAB] = 30 mM in an aqueous NaHCO_3_/Na_2_CO_3_ buffer with pH = 10.5 and *I* ≈ 0.25 M. Dark spots correspond to ice crystals. The mesh of the grid used as sample holder was masked. Original cryo-TEM images can be found in the ESI (Fig. SI1†).

A confirmation of WLMs was obtained from cryo-transmission electron microscopy (cryo-TEM) images of a solution containing [Blue] = 10 mM and [DTAB] = 30 mM. Two images of the same solution at different locations are shown in Fig. 3. Thin, entangled WLMs with lengths of several hundred nanometers are clearly visible.

The formation of WLMs is likely caused by successive growth upon addition of Blue to DTAB. To investigate this mechanism further and to obtain ensemble-average information on the morphology of Blue–DTAB assemblies in solution, SANS was performed on samples with compositions indicated by white dots in Fig. 2(a).

### Small-angle neutron scattering

**Fig. 4:**
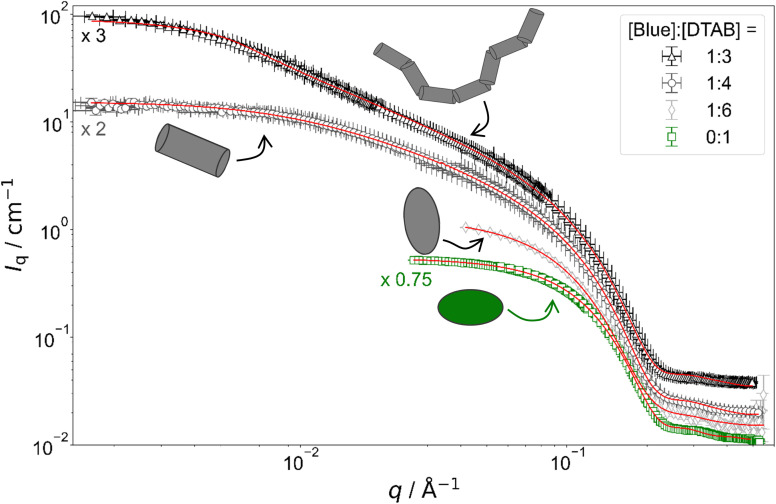
Full contrast SANS curves of solutions containing [DTAB] = 30 mM and varying concentrations of Blue in an aqueous NaHCO_3_/Na_2_CO_3_ buffer with pD = 10.7 and ionic strength *I* ≈ 0.25. Red lines display form factor fits according to the model of flexible cylinders with elliptical cross section^36^ for [Blue] : [DTAB] = 1 : 3, cylinders with elliptical cross section^37^ for [Blue] : [DTAB] = 1 : 4, triaxial ellipsoids^38^ for [Blue] : [DTAB] = 1 : 6 and oblate spheroids^37^ including a structure factor according to Hayter and Penfold in the rescaled mean spherical approximation^39–41^ for [Blue] = 0 mM.

**Fig. 5:**
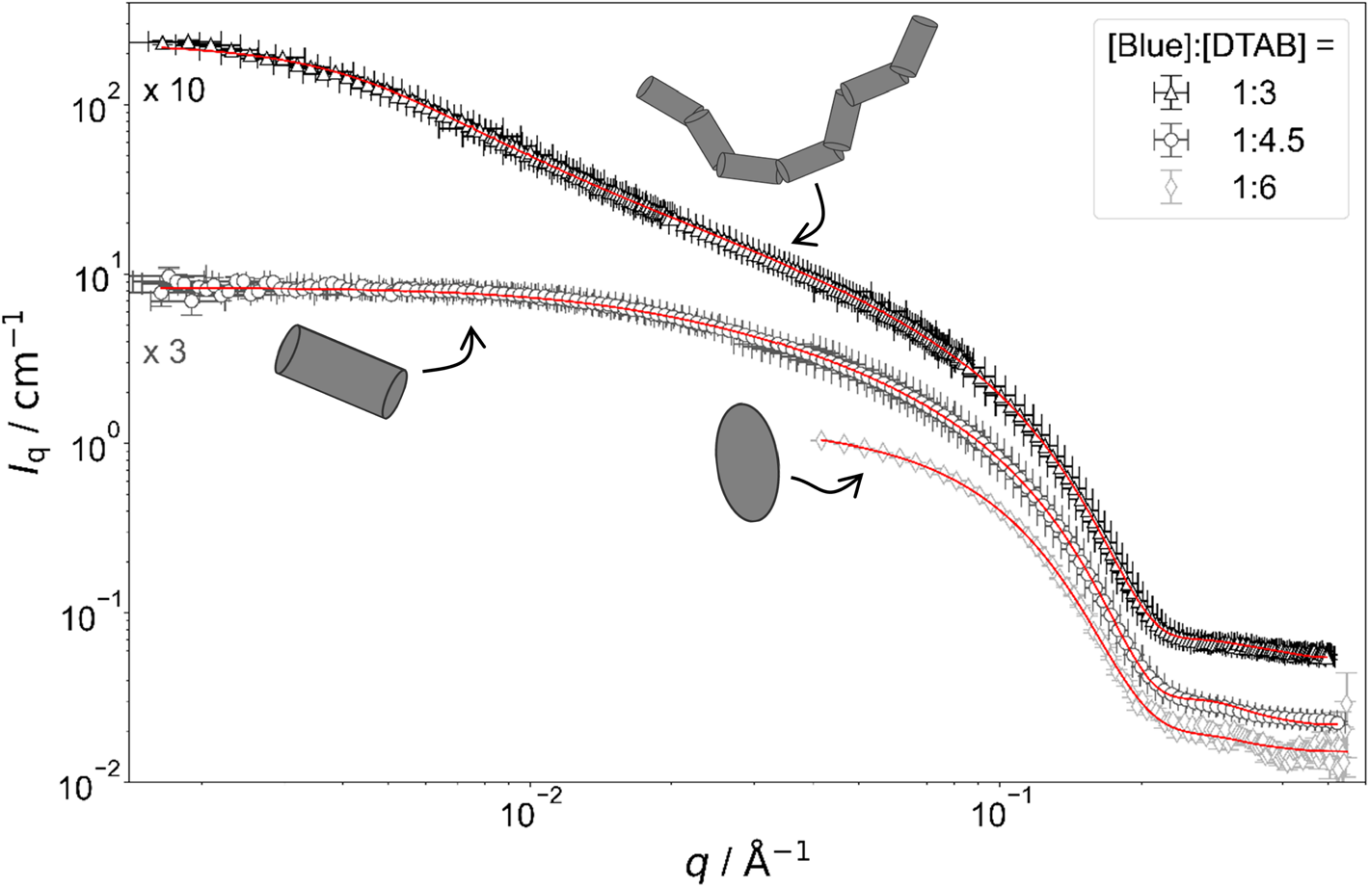
Full contrast SANS-curves of solutions containing [Blue] = 5 mM and varying concentrations of DTAB in an aqueous NaHCO_3_/Na_2_CO_3_ buffer with pD = 10.7 and ionic strength *I* ≈ 0.25. Red lines display form factor fits according to the model of flexible cylinders with elliptical cross section^36^ for [Blue] : [DTAB] = 1 : 3, cylinders with elliptical cross section^37^ for [Blue] : [DTAB] = 1 : 4.5 and triaxial ellipsoids^38^ for [Blue] : [DTAB] = 1 : 6. The latter is also shown in Fig. 4 and is displayed here for reference.

Fig. 4 shows full contrast SANS curves obtained from solutions containing [DTAB] = 30 mM and different concentrations of Blue. Similarly, Fig. 5 shows full contrast SANS curves obtained from solutions containing a constant concentration of [Blue] = 5 mM and varying concentrations of DTAB. First assessments of SANS curves will consider the molar ratio between Blue and DTAB ([Blue] : [DTAB]). From both figures a shift of the Guinier plateau to lower values of the magnitude of the scattering vector *q* with increasing [Blue] : [DTAB] is evident. This indicates an increase in the radius of gyration of the scatterers and therefore an increase in assembly size.^42^ Furthermore, the appearance of a power law of *I*_q_ ∼ *q*^−1^ in the mid-*q* region with increasing [Blue] : [DTAB] suggests a morphological transition from spherical to cylindrical structures.^7,43,44^ The SANS curves of samples containing Blue and DTAB at a molar ratio of 1 : 3 furthermore show a crossover region between *I*_q_ ∼ *q*^−1^ and a power law *I*_q_ ∼ *q*^−*x*^ with an exponent *x* > 1. This likely results from the appearance of flexibility upon elongation of the cylindrical structures. A power law behaviour of *I*_q_ ∼ *q*^−2^ is characteristic for the random walk configuration (*θ* solvents) and *I*_q_ ∼ *q*^−5/3^ is characteristic for SANS curves from polymer chains in the self-avoiding random walk configuration (good solvents).^36,44^ Once length scales probed by *q* approach the persistence length of those chains, a transition to *I*_q_ ∼ *q*^−1^ is observed.^36^ At higher *q*, a *I*_q_ ∼ *q*^−4^ decay is observed for all [Blue] : [DTAB] ratios due to the finite cross section of all micelles.^4,36^

To quantitatively estimate the size of Blue–DTAB assemblies, SANS curves were fitted with appropriate form factor models suggested by the previous qualitative discussion. The scattering length density SLD_1_ of Blue–DTAB co-assemblies was fitted to account for SLD changes, which are introduced by a variation in the composition of such assemblies through variation of sample composition. The SLD of the solvent was kept constant and the volume fraction of scatterers was calculated as indicated in the section on Data analysis. Fitted SLDs (ESI, Table SI6†) are reasonable for the investigated system and will not be discussed further. Form factor models and resulting size parameters are displayed in Table 1 and will be discussed in the following.

**Table 1:** Size parameters obtained from fitting full contrast SANS curves of solutions containing Blue and DTAB at varying concentrations in an aqueous NaHCO_3_/Na_2_CO_3_ buffer with pD = 10.7 and *I* ≈ 0.25 M at 25 °C. SANS curves and fits are visualized in Fig. 4 and 5. Except for the DTAB solution without Blue, no structure factor was used during fitting. For fitting the SANS curve from the 30 mM DTAB solution, a structure factor according to Hayter and Penfold in the rescaled mean spherical approximation was used^39–41^

[Blue]/mM	[DTAB]/mM	Form factor model	Cross section radii		Length *L*/Å
			*r*_minor_/Å	*r*_major_/Å	
0	30	Spheroid^37^ (oblate)^a^	14.048 ± 0.008	22.357 ± 0.009	
5	30	Ellipsoid^38^ (triaxial)^b^	15.3 ± 0.2	22.0 ± 0.3	32.0 ± 0.3
7.5	30	Cylinder with elliptical cross section^37^^,^^c^	14.336 ± 0.009	21.19 ± 0.04	*L̄* = 148 ± 1
					*σ*/*L̄* = 0.95
10	30	Flexible cylinder with elliptical cross section^36^^,^^d^	14.046 ± 0.008	21.15 ± 0.04	*L* = 1000 ± 6
					*l*_p_ = 300 ± 3
5	22.5	Cylinder with elliptical cross section^37^^,^^c^	14.78 ± 0.02	21.21 ± 0.06	*L̄* = 73.4 ± 2
					*σ*/*L̄* = 0.95
5	15	Flexible cylinder with elliptical cross section^36^^,^^d^	13.92 ± 0.02	21.19 ± 0.07	*L* = 1705 ± 12
					*l*_p_ = 209 ± 1

a For an oblate spheroid *r*_minor_ corresponds to the polar radius and *r*_major_ to the equatorial radius.

b For a triaxial ellipsoid *r*_minor_, *r*_major_ and *L* correspond to the radii of three semi-axes with *r*_minor_ < *r*_major_ < *L*.

c A Schulz distribution in cylinder length was assumed. *L̄* is the number-average cylinder length and *σ* its root-mean-square deviation.

d For a flexible cylinder, *L* denotes the contour length and *l*_p_ its persistence length.

#### DTAB micelles

The size and structure of pure DTAB micelles in various solvents was reported in previous literature. Bergström and Pedersen studied the structure of DTAB micelles in aqueous NaBr solutions at 40 °C using SANS.^39^ Dependent on the ionic strength of the solvent, SANS curves were best described using either the form factor of an oblate spheroid or a triaxial ellipsoid form factor considering intermicellar interactions with a structure factor according to Hayter and Penfold in the rescaled mean spherical approximation.^40,41^ The formation of oblate spheroidal micelles with a polar radius of 14 Å and an equatorial radius of 24 Å in a 0.2 M NaBr solution was suggested. As this solvent ionic strength of *I* = 0.2 M is similar to the ionic strength *I* ≈ 0.25 M of the NaHCO_3_/Na_2_CO_3_ buffer used as a solvent in the present work, the same form factor model was used to fit the SANS curve resulting from a 30 mM DTAB solution (Fig. 4). Micelles with a polar radius of 14 Å and an equatorial radius of 22 Å were found, which is comparable to Bergström's and Pedersen's results.

The use of a structure factor significantly improved the fit quality for the SANS curve from pure DTAB solution, even though no obvious correlation peak was observed. The absence of a distinct correlation peak results from screening of the surface charges of DTAB micelles by buffer salt.^45–47^ SANS curves from solutions containing Blue and DTAB also did not show a correlation peak. In addition to that, these SANS curves were sufficiently well described using only a form factor and no structure factor, which indicates negligible intermicellar interactions and ordering. This likely results from further screening of DTAB head group charges due to electrostatic interactions with the oppositely charged dye molecule.^47^

#### Ellipsoidal Blue–DTAB micelles

The SANS curve of the sample containing [Blue] = 5 mM and [DTAB] = 30 mM (ratio 1 : 6) is well described using the form factor of a triaxial ellipsoid. This resembles observations of Bergström and Pedersen who showed that with increasing ionic strength of the solution by NaBr addition, oblate spheroidal micelles turn into triaxial ellipsoids.^39^ However, the main reason for the morphological transition observed here is likely not the increase of solution ionic strength by ∼2% upon addition of 0.005 M of the acid Blue to the buffer with *I* ≈ 0.25 M. The adsorption of Blue on the micellar surface or even insertion into the micelle close to the surfactant head group results in more efficient screening of micellar surface charges and is a more plausible explanation for the observed behaviour.^4,47^ Furthermore, form factor fits (Table 1) indicate a strong similarity between the radius of the smallest semi-axis of Blue–DTAB triaxial ellipsoidal micelles (*r*_minor_ = 15.3 Å) and of the polar radius of oblate spheroidal DTAB micelles (*r*_minor_ = 14.0 Å) as well as the radius of the medium semi axis of the Blue–DTAB triaxial ellipsoidal micelles (*r*_major_ = 22.0 Å) and the equatorial radius of DTAB micelles (*r*_major_ = 22.4 Å). This could point towards an uniaxial growth of these micelles.^4^

#### Cylindrical Blue–DTAB micelles

SANS curves of samples containing [Blue] = 5 mM with [DTAB] = 22.5 mM (ratio 1 : 4.5) and [Blue] = 7.5 mM with [DTAB] = 30 mM (ratio 1 : 4) are well described using either the form factor of a cylinder with elliptical cross section or the form factor of a cylinder with circular, polydisperse cross section. The application of the model of a cylinder with elliptical cross section resulted in a better fit of experimental data. Furthermore, resulting cross section radii agree well with the minor and the medium semi-axes (*r*_minor_ and *r*_major_) of Blue–DTAB triaxial ellipsoidal micelles ([Blue] = 5 mM, [DTAB] = 30 mM, Table 1). Therefore, cylindrical Blue–DTAB assemblies likely exhibit an elliptical cross section. This assessment is supported by several sources in which micellar growth by hydrotrope or surfactant addition was studied.^4,13,47^ Among them, Bergström and Pedersen used a form factor model with elliptical cross section to describe mixed micelles of DTAB and sodium dodecyl sulfate (SDS).^13^ Furthermore, Hassan *et al.* suggested that the polydispersity obtained for the circular cross-section radius of cylindrical hydrotrope–SDS micelles likely results from an effective elliptical cross section of those micelles.^47^ Whereas cross section radii of Blue–DTAB assemblies remain approximately similar for all sample compositions (Table 1), cylinder lengths vary and show pronounced polydispersity. This is not unlikely and was already observed by Bergström and Pedersen for DTAB-SDS mixed micelles with similar morphologies.^13^ They considered the polydispersity in cylinder length by assuming a broad Schulz distribution. A similar strategy was applied for fitting SANS curves from Blue–DTAB solutions with the form factor model of cylinders with elliptical cross section. Herein, the length of cylindrical micelles was assumed to be distributed according to a Schulz distribution. Its relative standard deviation *σ*/*L̄* was fixed to *σ*/*L̄* = 0.95, where *L̄* is the number averaged contour length and *σ* the standard deviation of the number frequency. The resulting fit parameters are displayed in Table 1 with fits being shown in Fig. 4 and 5.

The length of cylindrical assemblies increases from 73.4 Å to 148 Å when decreasing the excess of DTAB from [Blue] : [DTAB] = 1 : 4.5 to [Blue] : [DTAB] = 1 : 4. Decrease of the DTAB excess to [Blue] : [DTAB] = 1 : 3 leads to further growth of cylindrical micelles and appearance of flexibility. Therefore, SANS curves of corresponding samples were described using a form factor model for flexible cylinders with elliptical cross section (Fig. 4 and 5).^36^ The appearance of almost identical cross section radii for all samples containing Blue and DTAB (Table 1) independent of length and applied form factor model confirms that micelles generally grow in only one dimension, leaving the elliptical cross section unaltered.^4^

#### Packing parameter

**Fig. 6:**
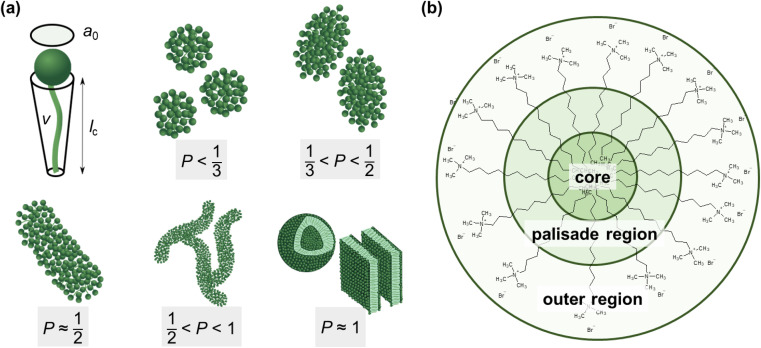
(a) Size parameters contributing to the packing parameter *P* of amphiphilic substances and schematic illustration of the relationship between the packing parameter and the morphology of assemblies.^48^ Spheres represent the hydrophilic surfactant head group whereas the hydrophobic tail is described by a line. (b) Classification of different regions of a DTAB micelle in water according to their hydrophilicity.^49,50^ The outer region consists of the positively charged DTAB head group and four neighbouring methylene groups, which are expected to interact with the surrounding water according to SANS investigations on pure DTAB micelles by Berr *et al.*^50^

The mechanism of the above-described morphological transition of DTAB micelles beginning with oblate spheroidal micelles *via* triaxial ellipsoidal micelles and rod-like cylinders to WLMs upon addition of Blue is easily explained by the concept of the packing parameter *P* for amphiphilic molecules:^48^4
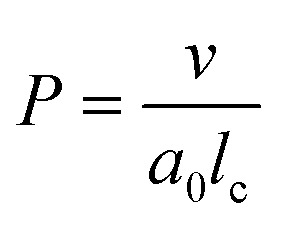
In eqn (4)*v* is the volume of the hydrophobic chain of the amphiphilic molecule, *l*_c_ its length and *a*_0_ the effective head group area occupied per molecule at the hydrocarbon–water interface.^48^ The packing parameter can be used to predict an assembly morphology based on these geometrical considerations. As shown in Fig. 6(a), low values 
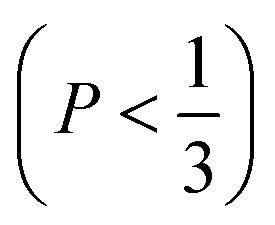
 imply spherical micelles, values of 
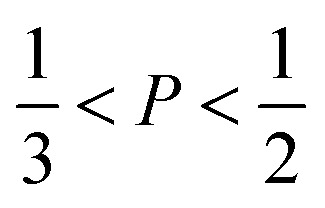
 point towards ellipsoidal micelles and for packing parameters around 
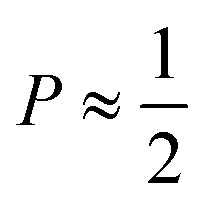
 the formation of cylindrical or rod-like micelles is favoured. Further increase results in the formation of various interconnected structures 
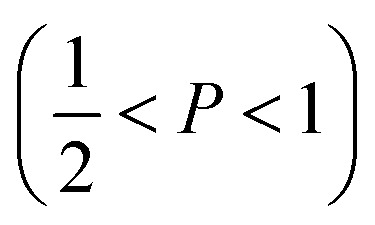
, vesicles and extended bilayers (*P* ≈ 1).^48^ This sequence of assembly morphologies goes along with a change in their spontaneous curvature from high for spherical aggregates to low for aggregates with locally flat interfaces such as bilayers.^3^

Based on the packing parameter it can be understood that intermolecular interactions between surfactant molecules and additives may change the morphology of surfactant micelles by changing the geometry of the amphiphile. In other words, the effective size of the hydrophobic and hydrophilic sections may be influenced by their rigidity and interactions such as hydrogen bonding or electrostatic interactions. Moreover, salt or hydrotrope addition usually reduces the effective head group size of ionic surfactant molecules due to charge screening and a concomitant reduction in electrostatic repulsion between head groups.^3,4^

Accordingly, the observed morphological transitions in solutions of Blue and DTAB can be reconciled with a reduction in DTAB head group size *a*_0_ due to shielding of electrostatic repulsion between positively charged DTAB head groups by negatively charged molecules of Blue. The reduction in *a*_0_ due to shielding of electrostatic repulsions must overcompensate an eventual increase of DTAB head group size caused by the geometrical inclusion of Blue into the head group layer to result in a net decrease of *a*_0_. This decrease of *a*_0_ leads to an increase of the packing parameter and explains the morphological transition from ellipsoidal to wormlike micelles upon successive addition of Blue. In addition to that, an increase of *P* can also be achieved if parts of the Blue molecules would contribute to the volume *v* of the hydrophobic surfactant chain. This would require a penetration of Blue into the palisade layer or core of the DTAB micelle. As Blue molecules carry a charge opposite to that of the DTAB head group and are well water soluble, they are expected to remain on the surface or outer layer of the micelle, rendering the reduction of effective head group size by charge screening the most likely reason for micellar growth. To confirm this hypothesis, it is necessary to locate Blue within the Blue–DTAB micelle. For this purpose, a combination of SANS contrast matching and NMR spectroscopy was used.

### Localization of Blue in Blue–DTAB micelles by means of contrast matching

SANS contrast matching gives access to one component of the mixed Blue–DTAB micelle by eliminating the contrast of the other component relative to the surrounding medium upon isotopic substitution. Within the investigations presented here, the contrast of DTAB relative to the aqueous solvent, *i.e.* NaHCO_3_/Na_2_CO_3_ buffer prepared in D_2_O, was eliminated by using deuterated d_25_-DTAB and d_34_-DTAB at a volume ratio of 46 : 54, which corresponds to the experimental match composition.

**Fig. 7:**
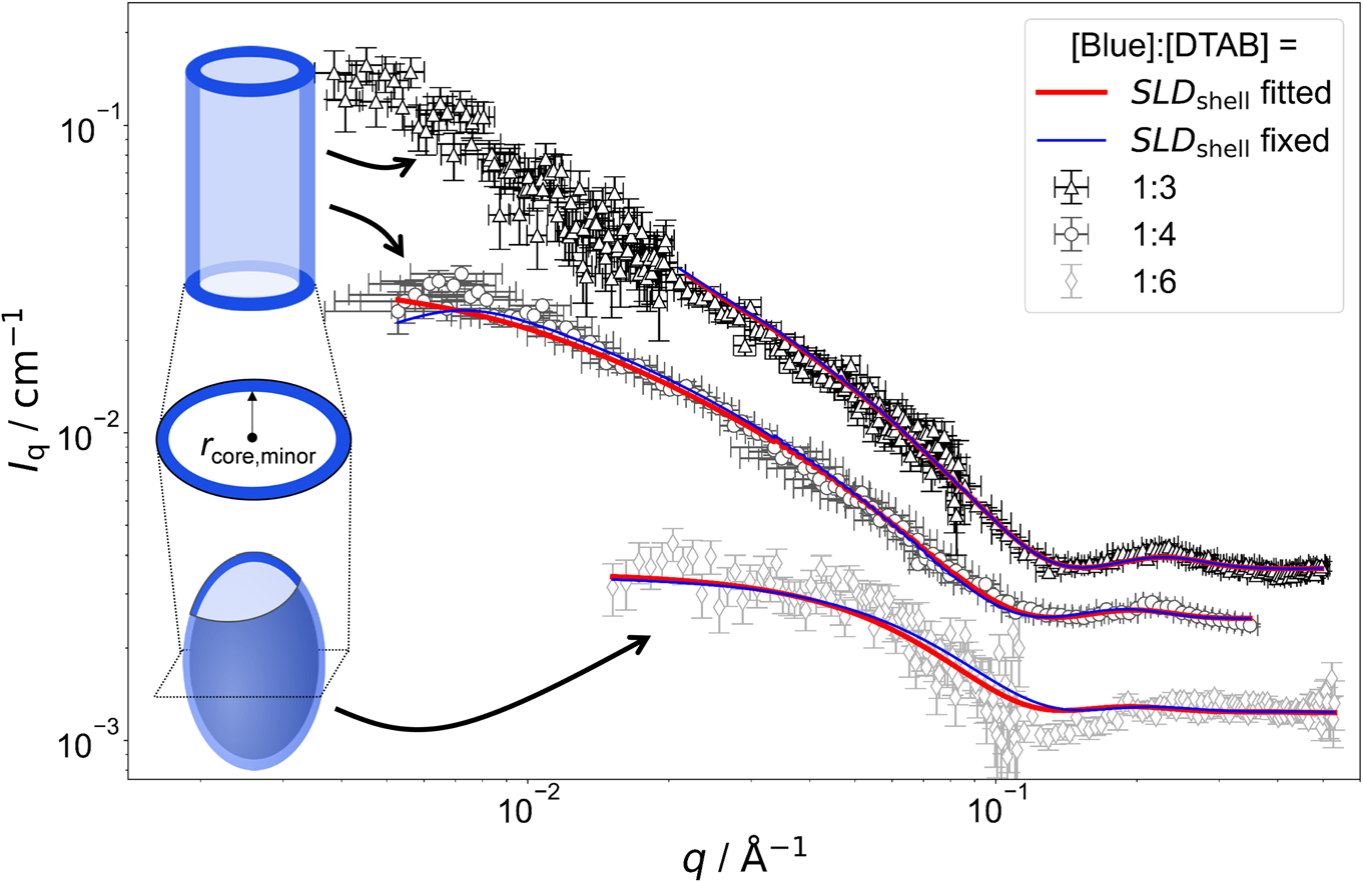
SANS curves of solutions containing 30 mM DTAB and varying concentrations of Blue with DTAB being a mixture of 46 vol% d_25_-DTAB and 54 vol% d_34_-DTAB corresponding to its experimental match composition. This means that the SLD of the surfactant mixture is the same as the SLD of the solvent, eliminating the contrast between the surfactant and the solvent. The solvent is a NaHCO_3_/Na_2_CO_3_ buffer (pD = 10.7, *I* ≈ 0.25 M) prepared in 100 vol% D_2_O. Measurements were performed at 25 °C. Red and blue lines display form factor fits according to the model of core–shell cylinders with elliptical cross section^51^ for [Blue] : [DTAB] ratios of 1 : 3 and 1 : 4 or the model of a triaxial core–shell ellipsoid for a [Blue] : [DTAB] ratio of 1 : 6. The fit displayed as red line was obtained when the SLD of the shell was fitted whereas the fit displayed as blue line was obtained while fixing the shell SLD.

**Fig. 8:**
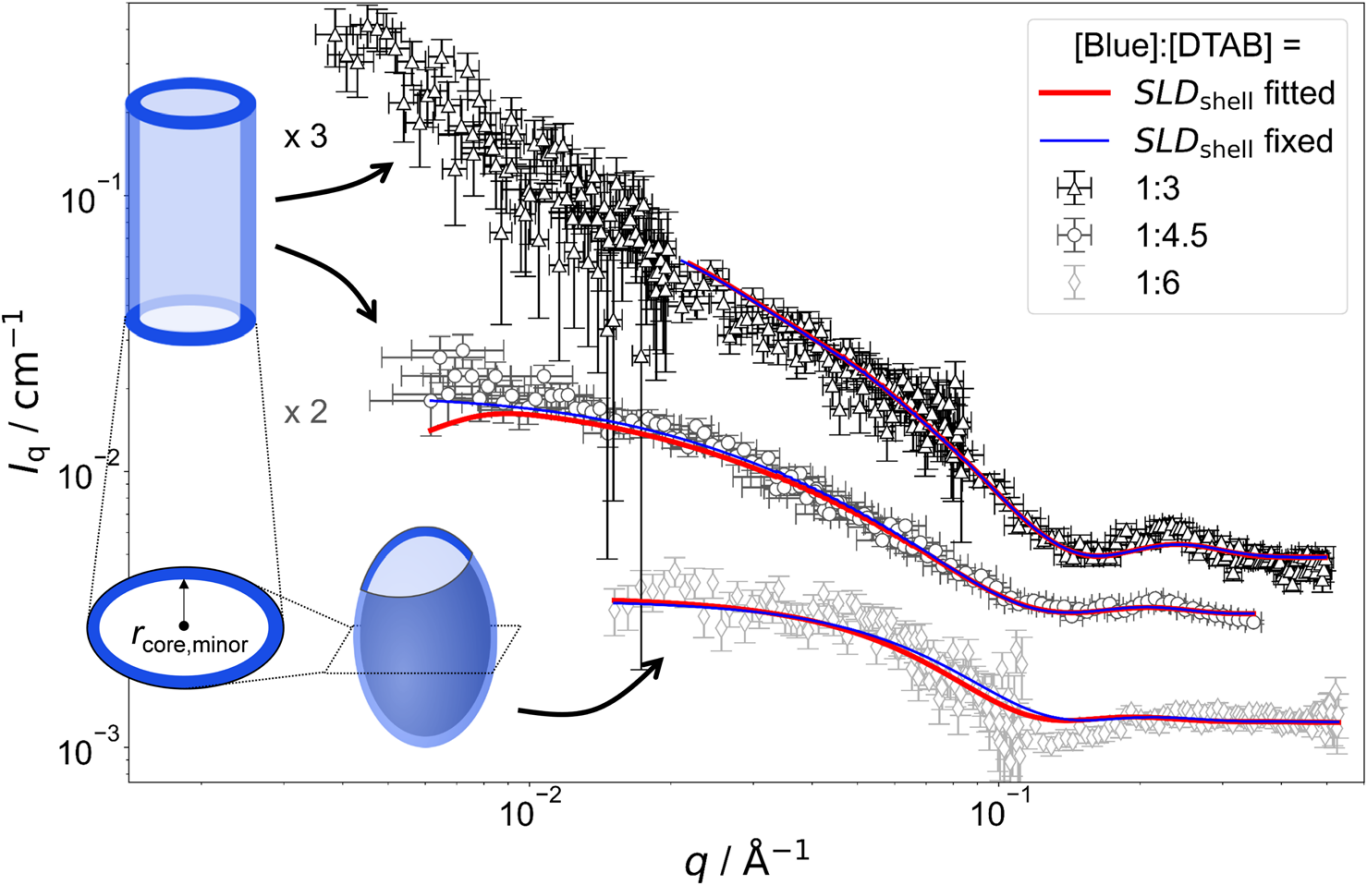
SANS curves of solutions containing 5 mM Blue and varying concentration of DTAB with DTAB being a mixture of 46 vol% d_25_-DTAB and 54 vol% d_34_-DTAB corresponding to its experimental match composition. This means that the SLD of the surfactant mixture is the same as the SLD of the solvent, eliminating the contrast between the surfactant and the solvent. The solvent is a NaHCO_3_/Na_2_CO_3_ buffer (pD = 10.7, *I* ≈ 0.25 M) prepared in 100 vol% D_2_O. Measurements were performed at 25 °C. Red and blue lines display form factor fits according to the model of core–shell cylinders with elliptical cross section^51^ for [Blue] : [DTAB] ratios of 1 : 3 and 1 : 4 or the model of a triaxial core–shell ellipsoid for a [Blue] : [DTAB] ratio of 1 : 6. The fit displayed as red line was obtained when the SLD of the shell was fitted whereas the fit displayed as blue line was obtained while fixing the shell SLD.

Fig. 7 shows SANS curves of solutions containing 30 mM DTAB and varying concentrations of Blue with DTAB being matched to the solvent. As a consequence, only scattering from Blue is observed. SANS curves from corresponding samples, based on the scattering from Blue and DTAB (full contrast), were shown in Fig. 4 and discussed before. Analogously, Fig. 8 and 5 show SANS curves from samples containing 5 mM Blue and varying concentrations of DTAB in DTAB-matched and full contrast mode respectively.

The form factor models used to describe experimental data are visually indicated next to the respective SANS curve in Fig. 7 and 8. Core-shell models were used in all cases with the scattering length density (SLD) of the core corresponding to the SLD of the solvent. This SLD was fixed to its theoretical value (6.376 × 10^−6^ Å^−2^) during fits. Two fitting strategies were employed: in a first attempt, the SLD of the shell was fixed to SLD_shell_ = 3.028 × 10^−6^ Å^−2^. This is the SLD of the one-fold deprotonated Blue based on its elemental composition and on the experimentally determined partial molar volume. In a second attempt the SLD of the shell was fitted. This permits a less dense shell of Blue due to hydration and proximity of the hydrophilic head groups of DTAB. For this reason, the latter strategy is more realistic and resulted into a similar or slightly better fit quality in most cases.^50^ However, results have to be evaluated with care as size parameters, such as shell thickness, depend on the SLD.^52^

Fitted curves are displayed together with experimental data in Fig. 7 and 8. Results related to the assembly cross section are displayed in Table 2. To reduce the number of fit parameters, parameters related to length and cross section anisometry were fixed to values obtained from fitting to corresponding full contrast data. The following details shall be explicitly outlined: owing to poor data quality in the low-*q* region, SANS curves containing [Blue] : [DTAB] = 1 : 3 for contrast matched DTAB were described using the form factor model of a rigid core–shell cylinder rather than a flexible core–shell cylinder. This does not depreciate the analysis, as the scattering signal in the evaluated high-*q* range does not carry information on flexibility of the overall assembly. Furthermore, information on overall assembly size were obtained from full contrast measurements. In addition to that, the evaluation of cross section dimensions should not be hampered by not considering low-*q* scattering. Therefore, lengths of core–shell cylinders used to describe SANS curves from DTAB contrast matched samples were fixed to values obtained from fitting corresponding full contrast SANS curves. Precise values can be found in the ESI (Table SI6†). In addition to the length parameter, the cross section anisometry of assemblies formed in DTAB contrast matched samples was fixed to the anisometry obtained from fitting full contrast SANS curves by fixing the ratio between the major and the minor core radius of the core–shell model (*r*_core,major_/*r*_core,minor_) to the ratio between *r*_major_ and *r*_minor_ of the respective full contrast model (Table 1). All parameters are summarized in the ESI (Table SI6†).

**Table 2:** Cross section size parameters of core–shell structures obtained from fitting SANS-curves from DTAB contrast matched solutions in comparison to the minor cross section radius *r*_minor_ obtained from fitting full contrast SANS-curves with a model assuming a homogenous SLD distribution. The radius of the shortest semi-axis of the triaxial ellipsoid form factor is given for the sample with [Blue] = 5 mM and [DTAB] = 30 mM and the minor radius of the elliptical cross section of the elliptical cylinder form factor in all other cases. The SLD of the core is equal to that of the solvent with SLD_core_ = SLD_solvent_ = 6.376 × 10^−6^ Å^−2^ for DTAB contrast matched structures^a^

[Blue]/mM	[DTAB]/mM	Full contrast	DTAB contrast matched, SLD_shell_ = 3.028 × 10^−6^ Å^−2^		DTAB contrast matched, SLD_shell_ fitted		
		*r*_minor_/Å	*r*_core,minor_/Å	*th*/Å	*r*_core,minor_/Å	*th*/Å	SLD_shell_/10^−6^ Å^−2^
5	30	15.3 ± 0.2	14.4 ± 0.6	1.6 ± 0.02	14.4 ± 0.6	4.7 ± 0.9	5.4 ± 0.2
7.5	30	14.336 ± 0.009	15.7 ± 0.2	0.874 ± 0.004	14.5 ± 0.2	2.1 ± 0.4	5.0 ± 0.2
10	30	14.046 ± 0.008	12.6 ± 0.2	1.28 ± 0.02	12.0 ± 0.8	2.4 ± 1.2	4.5 ± 0.3
5	22.5	14.78 ± 0.02	14.7 ± 0.3	0.748 ± 0.006	13.3 ± 0.4	3.3 ± 0.5	5.6 ± 0.2
5	15	13.92 ± 0.02	11.7 ± 0.3	1.41 ± 0.02	10.9 ± 0.5	3.5 ± 0.8	4.9 ± 0.3

a *r*_minor_ – minor radius of the elliptical cross section obtained from fitting full-contrast SANS curves, *r*_core,minor_ – minor radius of the elliptical cross section of the core obtained from fitting SANS-curves of samples, where DTAB was contrast matched, with a core–shell model, *th* – shell thickness, SLD_shell_ – scattering length density of the shell.

As many size parameters were adapted from fits to full contrast SANS curves, only the minor core radius *r*_core,minor_, the shell thickness (*th*) and, dependent on the employed strategy, SLD_shell_ needed to be fitted. To this end, two strategies were applied. In one strategy, SLD_shell_ was fixed and in the other strategy, SLD_shell_ was fitted. Table 2 displays only these parameters in comparison to *r*_minor_ obtained for the elliptical cross section from fitting full contrast SANS curves.

Comparing the results of both fitting strategies based on core–shell structures leads to two main observations: (1) shell thicknesses *th* are larger when SLD_shell_ was fitted and (2) fitted SLD_shell_ are higher than the SLD of negatively charged Blue, leading to lower contrast relative to the solvent. Both observations are easily explained by considering that the shell does not solely consist of a dense layer of Blue, but also solvent molecules and head groups from the SLD-matched DTAB. This leads to an increase in SLD and, as shell thickness and SLD correlate, a thicker shell.^52^ As fitting the SLD_shell_ resulted in more realistic thicknesses (>1 Å) compared to fitting with the SLD_shell_ being kept constant, further discussion will be based on the fits where SLD_shell_ was fitted.

From Table 2 it can be seen, that the cross section core radius of the core–shell structure *r*_core,minor_ is smaller than *r*_minor_ from analysis of full contrast curves in most cases except for the sample with [Blue] = 7.5 mM and [DTAB] = 30 mM. However, *r*_core,minor_ and *r*_minor_ agree within uncertainty of the values for this sample. The relation *r*_core,minor_ < *r*_minor_ implies that the “shell” containing Blue molecules extends into the DTAB micelle. Furthermore, the sum of *r*_core,minor_ and *th*, corresponding to the total minor radius of the elliptical cross section for the core–shell structure, is similar and systematically bigger than *r*_minor_ for all samples. The relation *r*_minor_ < *r*_core,minor_ + *th* implies that the shell likely ranges into the surrounding medium. Core radii and shell thicknesses vary only insignificantly with varying sample composition and therefore do not allow to discern any trends concerning the penetration depth of Blue into the DTAB micelle as a function of [Blue] : [DTAB] ratio.

As a major result, SANS based on contrast matching successfully demonstrates that Blue molecules are located close to the DTAB head groups in the Blue–DTAB co-assembly. From the presented results it is reasonable to further conclude that at least a part of the Blue molecules are localized next to the positively charged DTAB head groups.

### NMR-spectroscopy

#### ^1^H-NMR spectroscopy

**Fig. 9:**
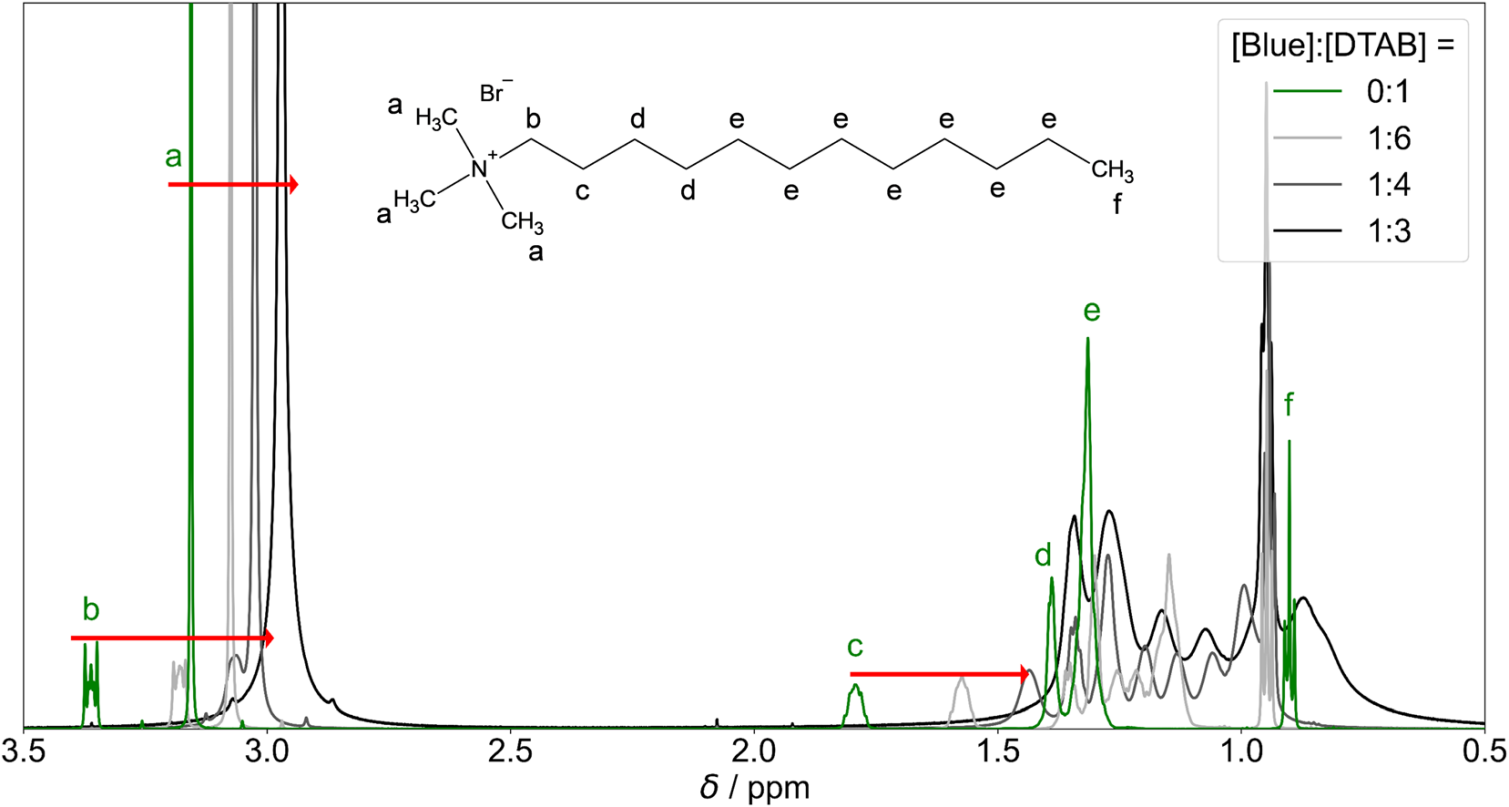
^1^H-NMR resonances of DTAB protons in solutions containing [DTAB] = 30 mM and varying concentrations of Blue. The green curve corresponds to the ^1^H-NMR spectrum of DTAB in the absence of Blue. Solutions were prepared in an NaHCO_3_/Na_2_CO_3_ buffer (pD = 10.7, *I* ≈ 0.25 M) in D_2_O. Red arrows indicate alterations in chemical shift of resonances a, b and c with increasing concentration of Blue.

NMR experiments were performed to further investigate intermolecular interactions between Blue and DTAB in solution. Fig. 9 shows resonance signals of DTAB in solutions containing [DTAB] = 30 mM and varying amounts of Blue. The ^1^H-NMR spectrum of pure DTAB is shown in green, along with the peak assignment.^53^ Peaks are narrow even though [DTAB] = 30 mM lays far above the cmc of 9 mM in the present solvent, where monomeric DTAB molecules and micellar aggregates exist in equilibrium. Narrow peaks for this system suggest rather loosely coupled DTAB molecules with a high degree of relative thermal mobility.^54^ From Fig. 9 it is visible that successive addition of Blue to a DTAB solution leads to an upfield shift of resonances associated with protons close to the DTAB head group (a, b, c, d) and splitting of the resonance signal of protons of type e, presumably due to slightly different shielding of these protons.^55,56^ The methyl group f experiences a small downfield shift. The upfield shift of most proton resonances is mostly caused by an increase in electron density upon addition of the negatively charged Blue. This results into shielding of protons close to the negative charge towards the external magnetic field.^17,57,58^ Furthermore, the ring current of aromatic moieties of the Blue molecule could affect the chemical shift of neighbouring protons. Depending on the localisation of these protons relative to the aromatic moiety, shielding or de-shielding can occur.^59^ However, the contribution of such ring current effects to changes in the chemical shift of DTAB proton resonances is assumed to be exceeded by the shielding due to the negative charge of Blue. Depending on the localisation of DTAB protons relative to the negatively charged phenolate group of Blue, some DTAB protons may experience stronger shielding and therefore a more pronounced upfield shift compared to others. This phenomenon may permit deductions on the localisation of Blue in DTAB micelles. To give an example, protons of type a or b experience a stronger change in their chemical shift upon Blue insertion than protons of type f, indicating that the negative charge of Blue is likely to be in direct proximity of type a or type b protons.^17,58^ Furthermore, the insertion of Blue causes a variation in electron density experienced by different protons of type e, resulting into splitting of the corresponding proton resonance.

The continuous upfield shift of NMR resonances with increasing [Blue] : [DTAB] ratio can be understood considering an equilibrium of multiple DTAB states with protons experiencing different local magnetic environments and therefore exhibiting different chemical shifts. Given a sufficiently fast exchange between these states, a single time-averaged spectrum is obtained.^53,55–57^ Increasing the [Blue] : [DTAB] ratio increases the fraction of DTAB molecules interacting with Blue and therefore the weighting of corresponding resonances for the time-averaged NMR spectrum, resulting into an upfield shift of time-averaged signals.

Apart from an upfield shift of most DTAB resonances, the addition of Blue results in peak broadening. This may be caused by two effects: (1) a penetration of Blue into the DTAB micelle likely decreases the mobility of DTAB molecules, favouring spin–spin relaxation and leading to peak broadening. (2) The formation of cylindrical micelles and micellar growth with increasing Blue : DTAB ratio results in a slowing of the end-over-end tumbling motion of rod-like micelles in the isotropic micellar phase. This causes a broadening of their proton resonances.^53,58,60^ The observations are therefore consistent with the previously made observations of aggregate growth with increasing dye-to-surfactant ratio.

**Fig. 10:**
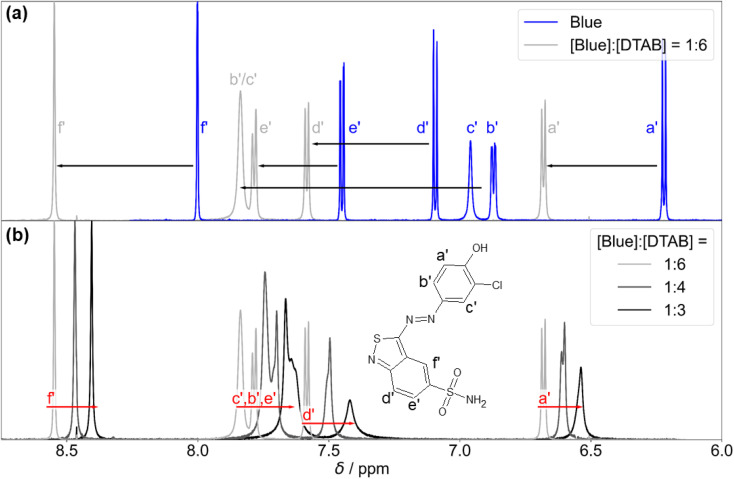
(a) ^1^H-NMR spectrum of Blue in its pure solution with [Blue] = 10 mM (blue line) compared to the ^1^H-NMR spectrum of Blue in presence of a 6-fold excess of DTAB (light grey line) with [Blue] = 5 mM and [DTAB] = 30 mM. Black arrows indicate alterations in the chemical shift of Blue proton resonances upon addition of DTAB. Resonances are labelled according to the designation of protons shown next to the chemical structure of Blue in (b). (b) ^1^H-NMR resonances of Blue in solutions containing [DTAB] = 30 mM and varying concentrations of Blue. Red arrows indicate alterations in ^1^H chemical shifts of Blue proton resonances in solutions containing DTAB and Blue with increasing concentration of Blue at constant DTAB concentration. Solutions were prepared in a NaHCO_3_/Na_2_CO_3_ buffer (pD = 10.7, *I* ≈ 0.25 M) in D_2_O.

Fig. 10(a) shows the ^1^H-NMR spectrum of Blue in the absence and in presence of DTAB. First and foremost, an obvious downfield shift of Blue proton resonances upon addition of DTAB is observed. This is likely caused by electrostatic interactions between Blue and DTAB and corresponding proximity of the positively charged DTAB head group to Blue, which results in a removal of electron density and subsequent de-shielding of Blue proton resonances upon DTAB addition.^17^

**Fig. 11:**
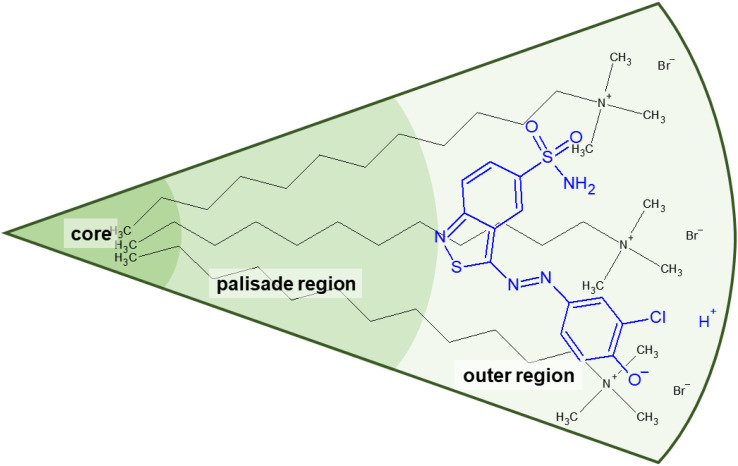
Suggested orientation of Blue in DTAB micelles according to the evaluation of DTAB-induced changes in chemical shifts of Blue proton resonances. The location was inferred from SANS contrast variation and evaluation of NOESY spectra.

Preliminary information on the orientation of Blue in DTAB micelles can be obtained by comparing the extent of downfield shifts observed for proton resonances from the two aromatic subunits of Blue upon addition of DTAB to a solution of pure Blue (Fig. 10(a)).^58^ The observed downfield shift is highest for resonances from protons b′ and c′ in meta-position to the phenolate group of the phenolic sub-unit with Δ*σ* of 0.97 ppm and 0.88 ppm for the sample containing [Blue] : [DTAB] = 1 : 6 compared to pure Blue. The signal position of proton a′ is affected less with Δ*σ* = 0.46 ppm, which is likely due to the delocalization of the negative phenolate charge into its *ortho*- rather than *meta*-position based on structural resonance formula.^61^ Therefore, a′ is likely less affected by the de-shielding effect caused by the proximity of the positively charged DTAB head group than b′ and c′. Proton resonances of the benzothiazole aromatic sub-unit were affected less leading to Δ*σ* = 0.49, 0.33 and 0.55 for d′, e′ and f′ respectively when comparing chemical shifts from the ^1^H-NMR spectrum of the sample with [Blue] : [DTAB] = 1 : 6 to pure Blue. Considering the stronger de-shielding for resonances of phenolic protons b′ and c′, the phenolate subunit is likely located closer to the positively charged DTAB head group than the benzoisothiazole subunit (Fig. 11). This is reasonable considering electrostatic attractions between the positively charged DTAB head group and the negatively charged phenolate. Furthermore, the mildly hydrophobic benzothiazole sub-unit is expected to penetrate into the DTAB micelle rather than protruding into the aqueous solvent. This hypothesis is examined by NOESY in one of the following sections.

Fig. 10(b) shows ^1^H-NMR resonance signals of Blue in solutions containing DTAB at a constant concentration of [DTAB] = 30 mM and varying concentrations of Blue. With increasing [Blue] : [DTAB] molar ratio, an upfield shift of all Blue proton resonances is observed. Two possible explanations for this observation exist, which are not mutually exclusive: at higher [Blue] : [DTAB] ratios more negatively charged Blue molecules are expected to interact with the positively charged DTAB head groups. This results into a higher density of Blue molecules in the DTAB head group region and subsequent increase in electron density. Therefore, the shielding of Blue protons located in that region successively increases with increasing concentration of Blue. Another explanation is related to the dynamic equilibrium present in micellar solutions: a ^1^H-NMR spectrum represents the time-average spectrum of all states of Blue present in solution given that the exchange between these states is fast enough.^53,55–57^ Therefore, an alternative possibility to explain resonance shifts would be a change in the molar ratio between these states with varying sample composition. Assuming the simplest case of two states of Blue, one interacting with DTAB and the other one being free in solution, the chemical shift of the time-averaged signal (*σ*) can be calculated from eqn (5).^55^

5 *σ* = *σ*_mic_*x*_mic_ + *σ*_free_(1 − *x*_mic_)

In eqn (5)*σ*_free_ is the chemical shift of a proton resonance of Blue in the free state, not interacting with DTAB (Fig. 10(a), blue line) and *x*_mic_ is the mole fraction of Blue interacting with DTAB. Furthermore, *σ*_mic_ is the chemical shift of a proton resonance of Blue interacting with DTAB, assuming that *σ*_mic_ does not change with varying [Blue] : [DTAB] ratio. However, as mentioned above, a change of *σ*_mic_ with an increasing number of Blue molecules interacting with DTAB micelles is possible. It is likely that both effects, variations in *x*_mic_ and changes in *σ*_mic_ contribute to changes in the chemical shift of Blue proton resonances with varying [Blue] : [DTAB] ratio.

Previous UV/vis spectroscopic investigations on the presented system indeed showed that the mole fraction of Blue molecules interacting with DTAB changes significantly from 0.95 for [Blue] : [DTAB] = 1 : 6 over 0.85 for [Blue] : [DTAB] = 1 : 4 to 0.73 for [Blue] : [DTAB] = 1 : 3. These values were calculated from the Blue–DTAB association constant of *K* = (4.7 ± 2) × 10^6^ L^3^ mol^−3^ for a stoichiometry of [Blue] : [DTAB] = 1 : 3.^33^ Accordingly, with decreasing DTAB excess, the fraction of Blue molecules interacting with DTAB micelles decreases.

#### NOESY

**Fig. 12:**
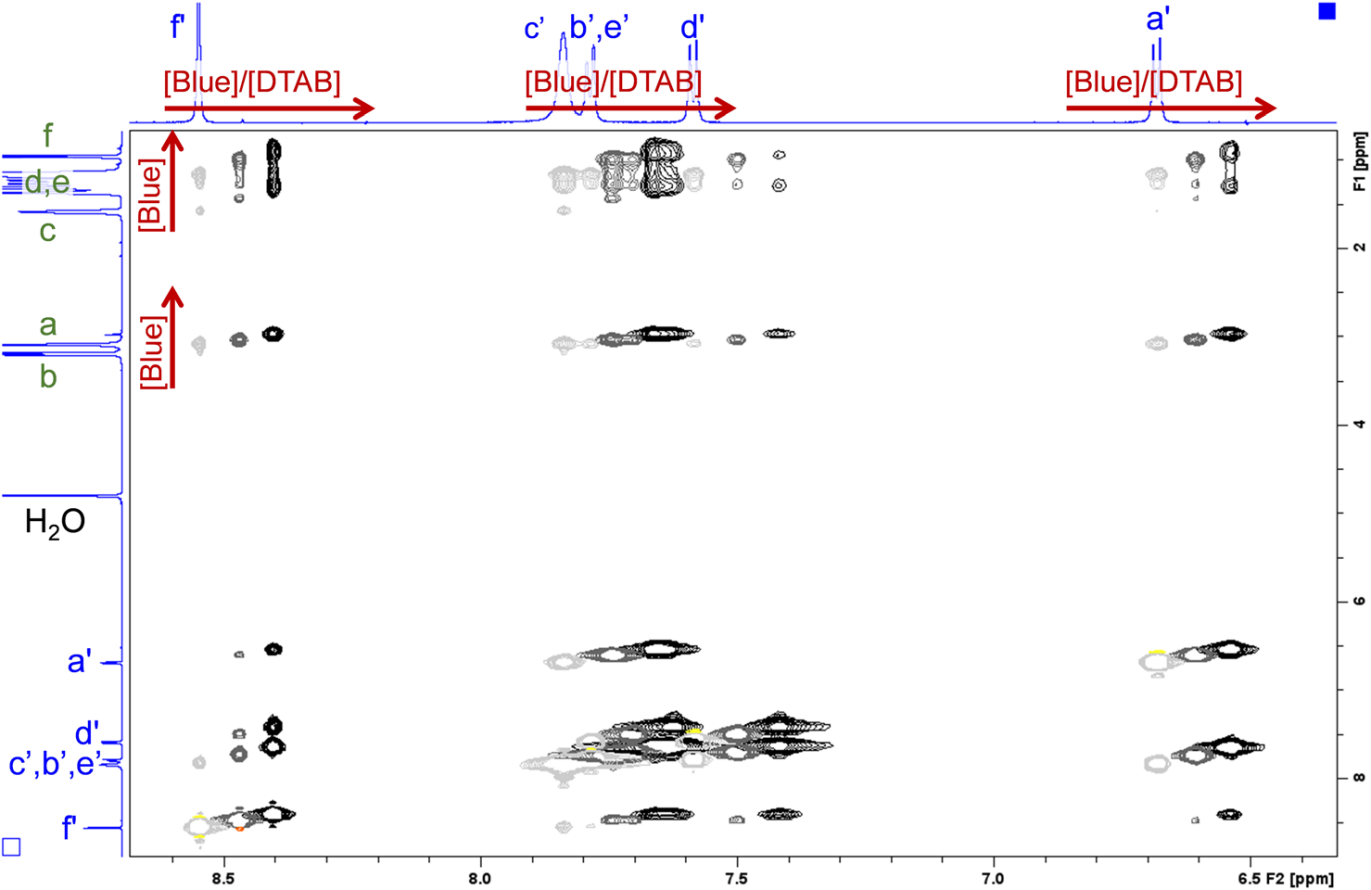
Section of NOESY spectra recorded from solutions containing [DTAB] = 30 mM and varying concentrations of Blue. The solvent is a NaHCO_3_/Na_2_CO_3_ buffer (pD = 10.7, *I* ≈ 0.25 M) prepared in D_2_O. Negative peaks are displayed in light grey, dark grey and black for samples containing [Blue] = 5 mM, 7.5 mM and 10 mM respectively. Positive peaks are displayed in yellow, orange and red for samples containing [Blue] = 5 mM, 7.5 mM and 10 mM respectively. Chemical shifts in the ^1^H-NMR spectrum induced by the increase of Blue concentration are indicated by red arrows. The ^1^H-NMR spectra of the sample containing [Blue] = 5 mM and [DTAB] = 30 mM are displayed on the side. Peak assignment was adopted from Fig. 9 and 10. The complete NOESY spectrum is shown in the ESI (Fig. SI9†).

Spatial proximity of protons up to a distance of about 5 Å can be studied by observing the Nuclear Overhauser Effect (NOE) with 2-dimensional NMR-spectroscopy by recording NOESY spectra.^17^Fig. 12 shows a relevant section of NOESY spectra from solutions containing [DTAB] = 30 mM and varying concentrations of Blue. As discussed above, ^1^H resonances are shifted relative to each other due to variation in the fraction of Blue interacting with DTAB, which makes it possible to display all three NOESY spectra in the same graph. Intensities of cross peaks are negative in all cases, which means that the zero-quantum transition *W*_0_ dominates over the double-quantum transition *W*_2_ causing the NOE.^17,18^ This points towards the presence of large aggregates.

Several cross peaks are visible and can be distinguished into (1) cross peaks between resonances of Blue, (2) cross peaks between resonances of DTAB and (3) cross peaks between resonances of Blue and resonances of DTAB. Cross peaks between resonances of Blue suggest spatial proximity among Blue molecules within Blue–DTAB co-assemblies. This proximity could be promoted by the formation of intermolecular π–π-stacking interactions between aromatic moieties of Blue. Such a mechanism was previously suggested for the interaction between oppositely charged dye and surfactant and would promote the formation of elongated, cylindrical assembly structures.^26^ Cross peaks between resonances of DTAB were not analysed due to strong overlap. Cross peaks between resonances of Blue and resonances of DTAB carry valuable information on the penetration of Blue into the DTAB micelle and are therefore discussed in the following.

Fig. 12 clearly shows several cross peaks between resonances of Blue and resonances of DTAB. Based on previous discussions, cross peaks between proton resonances arising from the DTAB trimethylammonium head group (a) and its geminal methylene group (b) and all Blue proton resonances are expected due to electrostatic interaction between the Blue and the DTAB head group. Furthermore, cross peaks are observed between all Blue proton resonances and the resonance arising from the methylene group in β-position to the ammonium ion (c). This is reasonable according to investigations made by Berr *et al.*, where the structure of DTAB micelles was studied using SANS contrast variation and who found, that the first four methylene groups of the DTAB alkyl chain are part of the hydrated shell of the DTAB micelle.^50^ For this reason, cross peaks should also be observed between Blue proton resonances and resonances of DTAB protons of type d. Unfortunately, an unambiguous assignment of DTAB alkyl chain resonances to corresponding protons is not possible due to peak broadening and shifts induced by the addition of Blue to DTAB solution. However, all Blue proton resonances show one or two additional cross peaks with resonances from protons in the DTAB alkyl chain. Due to the strongly upfield shifted spectrum of DTAB in Blue–DTAB solutions compared to pure DTAB solution, these cross peaks are attributed to the spatial proximity of Blue-protons and the four DTAB protons of type d. As discussed before, the strong upfield shift of these resonances results from an increase in electron density and subsequent shielding of proton resonances due to the interaction of DTAB with negatively charged Blue.^17^ Finally, the observation of cross peaks between DTAB proton resonances and all Blue proton resonances confirms the penetration of Blue molecules into DTAB micelles rather than a part of the Blue molecules sticking out of the micelles (Fig. 11).

To summarize results from the NMR-spectroscopic study, Blue was found to penetrate into the hydrated part of the DTAB-micelle, which corresponds to the Stern layer or outer region (Fig. 11).^49,50^ This outer region includes the trimethylammonium head group and the first four methylene groups of the DTAB alkyl chain.^50^ The shift of all ^1^H-NMR resonance signals upon variation of the [Blue] : [DTAB] ratio is likely caused by two effects: (1) the existence of various states of Blue and DTAB, *i.e.* Blue molecules interacting with DTAB micelles and Blue molecules being dissolved as single molecules in solution, generates a time-average resonance due to fast exchange between these two states. This resonance shifts with an increasing or decreasing fraction of Blue molecules interacting with DTAB. (2) A change in Blue- and DTAB-resonances due to continuous shielding or de-shielding effects upon variation of the composition of Blue–DTAB micelles.

## Conclusion

The co-assembly of the azo dye Blue and the cationic surfactant DTAB in an alkaline NaHCO_3_/Na_2_CO_3_ buffer with ionic strength of *I* = 0.25 M was investigated in detail. Small-angle neutron scattering clearly showed that addition of Blue in successive steps induces a uniaxial growth of pure, oblate spheroidal DTAB micelles to prolate ellipsoidal micelles and further on to cylinders and WLMs.

Using SANS contrast matching, Blue was observed to be located close to the DTAB head groups in all cases. These results were confirmed and extended by 2-dimensional NOESY. NOE dipolar coupling occurs between protons of Blue and DTAB alkyl chain protons up to the 4^th^ methylene group, thereby showing that the penetration boundary of Blue molecules reaches the inner boundary of the outer layer (Fig. 6). Furthermore, ^1^H-NMR spectroscopy shed light on the orientation of Blue in the DTAB micelle by the change of chemical shift of Blue proton resonances upon addition of DTAB: according to this observation, the phenolate group of Blue is expected to be located closer to the DTAB head group than the benzoisothiazole aromatic subunit.

The localization of Blue in the DTAB micelle reveals the reason for micellar growth upon addition of Blue. Micellar growth, which correlates with an increase of the packing parameter, can be caused by: (1) a reduction in the effective surface area per DTAB molecule or (2) an increase of the DTAB hydrophobic chain volume. Following the localisation of Blue close to the surfactant head group, an increase in the DTAB hydrophobic chain volume upon addition of Blue may be ruled out as the principal cause for the ellipsoid-to-cylinder transition. Therefore, Blue addition must cause a reduction of the effective surface area per DTAB molecule. This is likely caused by partial neutralisation of the positive DTAB head group charge upon interaction with negatively charged Blue, which overcompensates an increase in the DTAB head group size due to inclusion of the Blue molecule.

The complementary use of SANS contrast matching and NMR-spectroscopy is powerful for the localization of solutes in surfactant micelles. Not only does each method reveal a set of highly relevant information, but also does their complementarity reduce doubts in data interpretation, which could be caused by ambiguities of results obtained by just one method. As the feasibility of performing SANS contrast matching experiments for the localization of solutes in small surfactant micelles was demonstrated, such use may be particularly helpful in systems, where NMR peak assignment is hampered by peak overlap or unexpected changes in chemical shifts.

## Conflicts of interest

There are no conflicts to declare.

## Supplementary Material

NA-005-D3NA00556A-s001
