# Supplementary material for: SANS contrast matching for the unambiguous localization of anionic dye in cationic surfactant micelles
Source: Nanoscale Adv. 2023 Sep 11;5(19):5367–84. doi: 10.1039/d3na00556a (PMC10521298; doi:10.1039/d3na00556a)
Supplement: NA-005-D3NA00556A-s001 [file NA-005-D3NA00556A-s001.pdf]

Electronic Supplementary Material (ESI) for Nanoscale Advances.
This journal is © The Royal Society of Chemistry 2023

Supporting Information
SANS contrast matching for the unambiguous localization of anionic dye in cationic surfactant micelles

Wenke Müller*[a], Ralf Schweins[a], Bernd Nöcker[b], Hans Egold[c], Yvonne Hannappel[d] and Klaus Huber[c]

a. Institut Laue-Langevin, DS/LSS, 71 Avenue des Martyrs, 38000 Grenoble, France.

b. KAO Germany GmbH, Pfungstädter Straße 98-100, 64297 Darmstadt, Germany.

c. Universität Paderborn, Warburger Straße 100, 33098 Paderborn, Germany.

d. Universität Bielefeld, Universitätsstrasse 25, 33615 Bielefeld, Germany.

Experimental

Density and partial molar volumes

Density measurements were performed using an Anton-Paar Density Meter (DMA 4500 M) requiring a sample volume of 1 mL. Density measurements were performed to make the calculation of partial molar volumes $V_m(c)$ of Blue in buffer possible. For this purpose, five solutions containing Blue concentrations between 1 mM (0.37 g L^{-1}) and 20 mM (7.39 g L^{-1}) were prepared in an $NaHCO_3$/Na_2CO_3 buffer (pD = 10.7, $I \approx 0.25$) in 100 % D_2O. The densities of these solutions as well as of the buffer were measured after equilibrating the temperature of each sample to 25 °C. This yielded sample densities (ρ) and the density of the buffer corresponding to the solvent (ρ_0).

First, the partial molar volume of Blue in each of the measured solutions was calculated according to eq (1).[1]

$$V_m(c) = \frac{1000 \cdot (\rho_0 - \rho)}{c \cdot \rho} + \frac{M}{\rho} \tag{1}$$

In eq (1) *c* is the mass concentration of Blue and *M* its molar mass. In the present case, partial molar volumes $V_m(c)$ did not depend on *c*. Therefore, the average of all determined $V_m(c)$ was calculated to obtain the partial molar volume of Blue. Further partial molar volumes were obtained from literature. Table SI1 summarizes partial molar volumes used for the calculation of scattering length densities (*SLD*) of assembly components and of assembly volume fractions, the latter being required as an input parameter in SasView.

Viscosity

The kinematic viscosity ν of a $NaHCO_3$/Na_2CO_3 buffer (pD = 10.7, $I \approx 0.25$ M) in D_2O was determined using an Ubbelohde viscosimeter. (Type 52710/I Schott, viscosimeter constant K = 0.009433 $mm^2\ s^{-2}$). Viscosity

measurements were performed at 25 °C. For calculating the dynamic viscosity η, the experimentally determined buffer density of $\rho_0 =$ 1.11437 g cm^{-3} at a temperature of 25 °C was used:

$$\eta = \nu \cdot \rho_0 \quad (2)$$

Dynamic light scattering

Light scattering measurements were performed on an ALV CGS-3 Compact Goniometer System (ALV GmbH, Langen, FRG) using a HeNe laser at a wavelength of 632.8 nm. Measurements were performed using cylindrical quartz glass cuvettes with an inner diameter of 1 cm. The temperature of the the sample in the toluene bath was controlled using a thermostat and set to 25 °C. Intensity correlation functions ($g^{(2)}(\tau)$) resulting from dynamic light scattering (DLS) were fitted with a mono exponential model according to the revised method of cumulants, which permits the inclusion of longer correlation times into the analysis as compared to the traditional method of cumulants.[2] An expansion up to the second cumulant was considered:

$$g^{(2)}(\tau) = B + \beta \cdot \exp\left(-2\Gamma\tau\right) \cdot \left(1 + \frac{\mu_2}{2!} \cdot \tau^2\right)^2 \quad (3)$$

B is commonly referred to as baseline or the long-time value of $g^{(2)}(\tau)$, β is a factor that depends on experimental geometry, Γ is the decay rate, μ_2 is the second-order cumulant and τ is the correlation time. An apparent diffusion coefficient D_{app}, which potentially depends on the scattering angle, can be calculated from the decay rate:[2]

$$\Gamma = D_{app} \cdot q^2 \quad (4)$$

q is the magnitude of the scattering vector, which is calculated from the scattering angle. If D_{app} shows an observable *q*-dependency, the diffusion coefficient *D* is then obtained from the intercept in a linear fit according to the dynamic Zimm equation:[3]

$$D_{app}(q^2) = D \cdot (1 + K \cdot q^2) \quad (5)$$

where *D* is the diffusion coefficient of the particle and *K* a factor depending on sample polydispersity, particle morphology and size. If the sample consists of monodisperse spheres, $K = 0$ and $D_{app}(q) = D$.

The hydrodynamic radius R_h of the particles or assemblies in solution is obtained from the Stokes-Einstein-equation:[3]

$$R_h = \frac{k_B \cdot T}{6 \cdot \pi \cdot \eta \cdot D} \quad (6)$$

Where k_B is Boltzmann's constant, *T* the temperature and η the dynamic viscosity of the solvent.

In the presented work, dynamic light scattering experiments were performed on solutions containing differently deuterated species of DTAB at a concentration of [DTAB] = 30 mM in a $NaHCO_3/Na_2CO_3$ buffer (pD = 10.7, $I \approx 0.25$ M) in D_2O. The viscosity of the buffer was measured according to the above-described procedure and amounts to $\eta = (1.157 \pm 0.002)$ cP at a temperature of 25 °C.

Scattering length densities

For the analysis of small-angle neutron scattering curves with form factor models, the volume fraction of the Blue-DTAB co-assembly was fixed to a value calculated from known partial molar volumes V_m and concentrations of assembling molecules. For this purpose, it was assumed that partial molar volumes estimated for the components remain unchanged while being incorporated into the assemblies and that all molecules participate in assembly formation, ignoring the existence of monomeric molecules below the critical micelle concentration (*cmc*) of these molecules. Given the neglect of the *cmc* and considering that molar volumes are likely to be slightly altered upon assembly formation, the presented strategy results in an estimation rather than a precise calculation of volume fractions. Nevertheless, it is good enough to facilitate reduction of the number of parameters during fitting, which by no means lowers the validity of the obtained size parameters. Above all, the scattering length density (*SLD*) was fitted in most cases.

Table SI1 summarizes partial molar volumes used for the calculation of assembly volume fractions and theoretical *SLD*s of molecules and structural units. The scattering length density *SLD* of a molecule can be calculated from the coherent scattering lengths b_j of its constituting atoms, their respective number n_j and the partial molar volume of the molecule V_m according to eq (7):[4]

$$SLD = N_A \cdot \frac{\sum_j n_j \cdot b_j}{V_m} \quad (7)$$

In eq (7) N_A is Avogadro's number in [mol^{-1}], the partial molar volume V_m is given in [cm^3 mol^{-1}] and scattering lengths b_j are given in [cm].

Table SI1: Partial molar volumes and scattering length densities of molecules and structural building blocks. Additivity of partial molar volumes was assumed. Concentration of buffer salts in the solvent: [$NaHCO_3$] = 0.0214 mol L^{-1}, [Na_2CO_3] = 0.0786 mol L^{-1}.

Molecule or structural units	V_m $cm^3\ mol^{-1}$	*SLD* 10^{-6} Å^{-2}
BlueH ($C_{13}H_9ClN_4O_3S_2$)	246.31[I]	3.004
Blue (deprotonated, $C_{13}H_8ClN_4O_3S_2$)	218.58[II]	3.028
CH_3	32.70[5]	-0.842
CD_3	32.70[III]	4.910
CH_2	16.20[5]	-0.309
CD_2	16.20[5]	7.430
$N(CH_3)_3Br$	91.81[IV,6,7]	0.160
$N(CD_3)_3Br$	91.81[III]	6.306
h_{34}-DTAB ($C_{15}H_{34}BrN$)	302.70[IV]	-0.224
d_{34}-DTAB ($C_{15}D_{34}BrN$)	302.70[IV]	6.817
$CH_3(CH_2)_{11}$	210.89[IV]	-0.392
$CD_3(CD_2)_{11}$	210.89[IV]	7.040
H_2O	18.07[8]	-0.558
D_2O	18.13[8]	6.358
$NaHCO_3/Na_2CO_3$ buffer in 100 vol% D_2O	18.04[V,7]	6.376[VI]
$NaHCO_3/Na_2CO_3$ buffer in 50 vol% D_2O and 50 vol% H_2O		2.918[VII]
$NaHCO_3/Na_2CO_3$ buffer in 100 vol% H_2O	17.98 [V,7]	-0.540[VI]

[I]Measured, [II]Calculated from measured data and $V_m(H^+)$ = -5.5 $cm^3\ mol^{-1}$ following additivity principles.[7,9], [III]Assumed to be the same as for the hydrogenated species., [IV]Calculated assuming additivity of molar volumes., [V]Calculated assuming additivity of molar volumes and considering buffer composition., [VI]Calculated considering buffer composition., [VII]Calculated considering *SLDs* of buffers containing 100 vol% D_2O and 100 vol% H_2O.

Cryo transmission electron microscopy

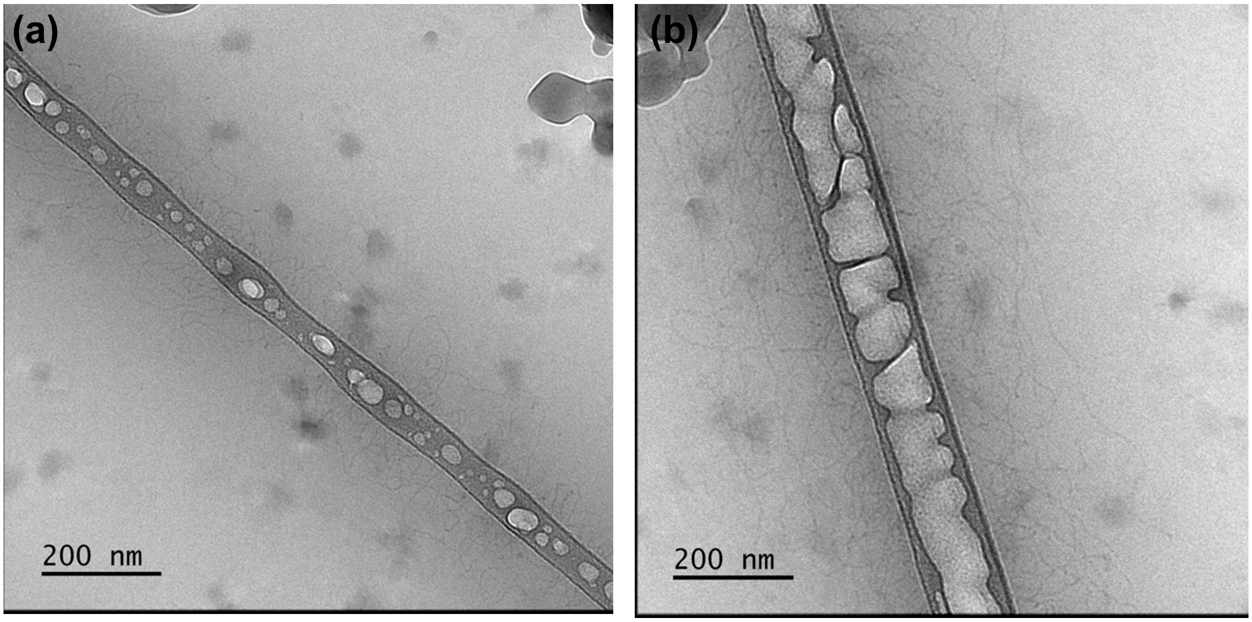

Figure SI1: Cryo TEM images of a solution containing [Blue] = 10 mM and [DTAB] = 30 mM in an aqueous $NaHCO_3/Na_2CO_3$ buffer with pH = 10.5 and $I \approx 0.25$ M.

Small-angle neutron scattering and assembly structure

Pure DTAB micelles

The size of pure DTAB micelles in the applied $NaHCO_3/Na_2CO_3$ buffer (pD = 10.7, $I \approx 0.25$ M) in D_2O at 25 °C was studied by recording SANS curves from solutions of three differently deuterated DTAB species, i.e. h_{34}-DTAB, d_{34}-DTAB and d_{25}-DTAB, with a total DTAB concentration of [DTAB] = 30 mM (Figure SI2).

Furthermore, a SANS curve was recorded from a solution containing DTAB with a concentration of [DTAB] = 30 mM and at match composition (46 vol% d_{25}-DTAB and 54 vol% d_{34}-DTAB) and is also shown in Figure SI2. The curve is lacking any *q*-dependence and thus confirms successful matching of the *SLD* of the DTAB mixture to the *SLD* of the solvent.

SANS curves from the other three samples were described using the product of a form- and a structure factor, as the use of a structure factor improved the fit in the low*q* regime. In all cases, a structure factor derived by Hayter and Penfold was used assuming a micellar charge of 15.[10,11] The assumption of a micellar charge of 15 resulted in a better fit compared to the assumption of a micellar charge of 10, 20, 30 or 50. It is emphasized, that the choice of a micellar charge within this range does not affect the resulting fit parameters (r_{eq} and r_p), as these parameters agree when rounded to the nearest integer (investigation not shown). The salt concentration was set to the ionic strength of the buffer (0.25 M) and the D_2O dielectric constant was set to 78.06 considering the measurement temperature of 25 °C.[12]

For describing SANS curves emerging from solutions of h_{34}-DTAB and d_{34}-DTAB, the form factor model of an oblate spheroid with homogeneously distributed scattering length density (*SLD*) was used.[13] For describing the SANS curve emerging from a solution of d_{25}-DTAB, a core-shell structure was employed.[14] Results from form factor fitting are displayed in Table SI2. The structure of DTAB micelles was previously studied in NaBr solutions, corresponding either to oblate spheroids of revolution or to triaxial ellipsoids

depending on the ionic strength of the solution.[15] The results from this reference agree with the dimensions of the DTAB micelles displayed in Table SI2.[15]

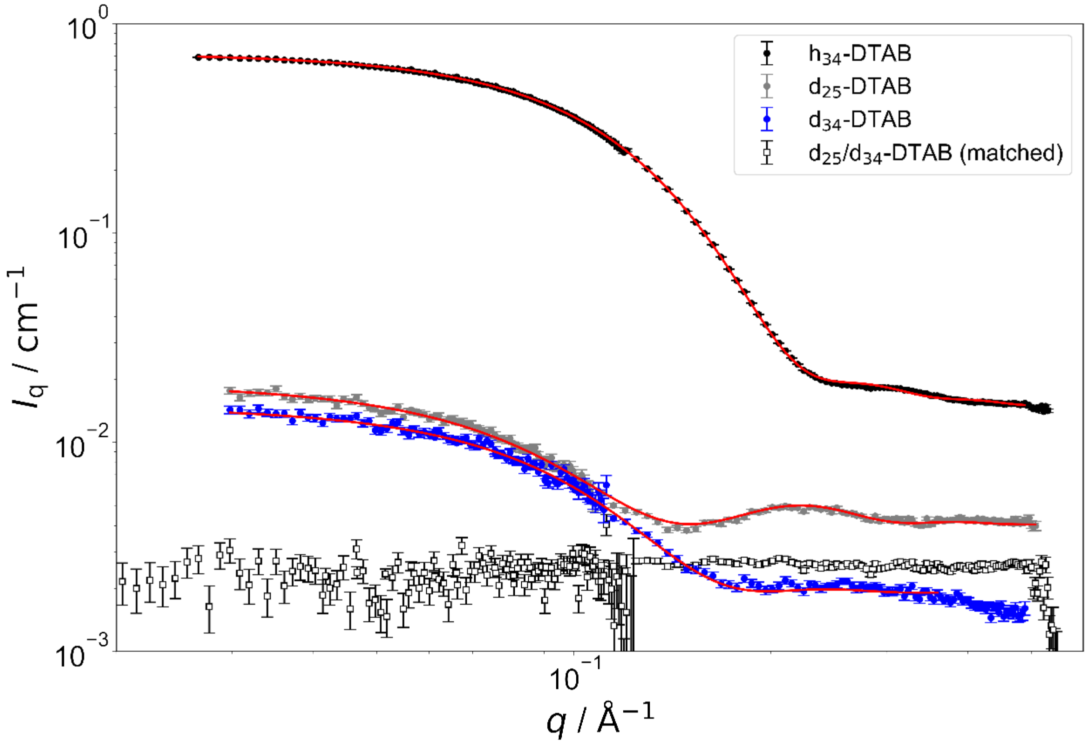

Figure SI2: SANS curves of solutions containing 30 mM DTAB with different degrees of deuteration in a $NaHCO_3/Na_2CO_3$ buffer with pD = 10.7 and ionic strength $I \approx 0.25\ M$. D_2O was used to prepare the buffer. From a solution containing 46 vol% d_{25}-DTAB and 54 vol% d_{34}-DTAB no *q*-dependent scattering is observed (□). Fits are shown as red lines and correspond to the product of the form factor of an oblate spheroid with a structure factor derived by Hayter and Penfold for h_{34}-DTAB and d_{34}-DTAB.[10,11] For d_{25}-DTAB, the product of the form factor of an oblate core-shell spheroid with the same structure factor was used.

Table SI2: Parameters resulting from fitting SANS curves of solutions containing differently deuterated DTAB species in an $NaHCO_3/Na_2CO_3$ buffer (pD = 10.7, $I \approx 0.25$ M) in D_2O and at a concentration of [DTAB] = 30 mM. The scattering length density of the solvent $SLD_{solvent}$ was fixed to $6.376 \cdot 10^{-6}$ Å^{-2}. For fitting SANS curves from solutions of h_{34}-DTAB or d_{34}-DTAB, the form factor model of an oblate spheroid was used. For fitting the SANS curve from a solution of d_{25}-DTAB, the form factor model of a core-shell oblate spheroid was used.

Surfactant	r_{eq} Å		r_p Å	*SLD* 10^{-6} Å^{-2}		χ^2
h_{34}-DTAB	22.357 ± 0.009		14.048 ± 0.008	-0.11 ± 0.04		12.667
d_{34}-DTAB	25.6 ± 0.7		19.3 ± 1.0	7.1 ± 0.2		1.4089
	th Å	$r_{core,eq}$ Å	$r_{core,p}$ Å	SLD_{core} 10^{-6} Å^{-2}	SLD_{shell} 10^{-6} Å^{-2}	χ^2
d_{25}-DTAB	7.1 ± 0.8	17.5 ± 0.6	11.9 ± 1	6.80 ± 0.08	5.1 ± 0.3	1.6331

r_{eq} – equatorial radius, r_p – polar radius, *SLD* – scattering length density of scatterer, χ^2 – chi square parameter divided by the number of datapoints, *th* – shell thickness, $r_{core,eq}$ – equatorial radius of the core, $r_{core,p}$ – polar radius of the core, SLD_{core} – scattering length density of the core, SLD_{shell} – scattering length density of the shell

However, Table SI2 reveals discrepancies between the radii of h_{34}-DTAB micelles and the radii of d_{34}-DTAB or d_{25}-DTAB micelles, obtained from form factor fits to corresponding SANS curves. A first attempt to explain these differences could be based on the occurrence of "isotope effects", i.e. different sizes of micelles being caused by different degrees of deuteration of the surfactant. Such effects were previously observed for alkyltrimethylammoniumbromide surfactants when changing solvent composition in terms of H_2O/D_2O ratio in the solvent.[5,16] Conversely, the use of differently deuterated species in the same solvent was previously not suspected to result in observable differences of assembly size and structure.[17] Furthermore, DLS data collected before the neutron experiment on the very same solutions do not suggest a change in size of DTAB micelles when varying its isotopic composition from h_{34}-DTAB over d_{25}-DTAB to d_{34}-DTAB. Correlation functions of solutions containing [DTAB] = 30 mM in the $NaHCO_3/Na_2CO_3$ buffer in D_2O overlap at all angles and are shown exemplarily for three angles in Figure SI3. Furthermore, fitting the correlation function with a single-exponential decay according to a revised method of cumulants (eq (3)) resulted in similar apparent diffusion coefficients D_{app} for solutions of all DTAB-species and at all probed angles (Figure SI4).[2] Finally, Table SI3 shows hydrodynamic radii R_h obtained for each DTAB-species. Apparent differences lay within the range of the error and micelles of h_{34}-DTAB, d_{25}-DTAB and d_{34}-DTAB are therefore of the same size according to dynamic light scattering measurements. In addition to the hydrodynamic radii R_h of DTAB micelles, Table SI3 also shows radii of gyration obtained from linearized Guinier analysis of corresponding SANS curves (Figure SI5). Radii of gyration also agree within experimental uncertainty, with an average value of R_g = 15.8 Å and a maximum deviation of ΔR_g = 1.7 Å.

It was previously suggested that small differences in scattering contrast ($\Delta SLD < 2 \cdot 10^{-6}$ Å^{-2}) do not permit an accurate size determination, referring to the thickness of the shell in a core-shell structure.[18] Considering the small difference in the scattering length densities (*SLD*s) between the solvent ($NaHCO_3/Na_2CO_3$ buffer in 100 vol% D_2O) and the trimethylammoniumbromide head group $N(CD_3)_3Br$ (Table SI*1*) of the d_{34}-DTAB molecule, this could be the case for d_{34}-DTAB. To verify this assumption, experiments with solvents containing varying H_2O/D_2O ratios would need to be performed and compared to isotope effects caused by the use of different solvents with small-angle X-ray scattering. Here, we abstained from such an analysis and the differences were attributed to uncertainties in the *SLD* values. This is backed by DLS investigations, which reveal the same hydrodynamic radius R_h for h_{34}-DTAB, d_{25}-DTAB and d_{34}-DTAB micelles thus discarding isotope effects. Furthermore, the dimensions of the h_{34}-DTAB micelle were used for further evaluation and discussion, as the highest SANS scattering contrast between surfactant micelle and solvent is obtained in this sample.

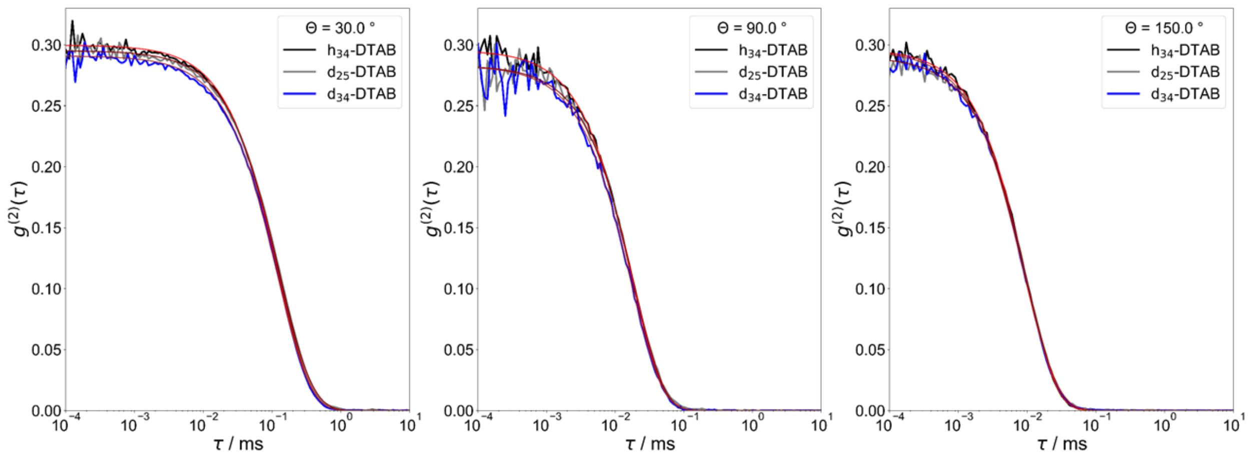

Figure SI3: Intensity correlation functions $g^{(2)}(\tau)$ obtained from DLS measurements with solutions containing h_{34}-DTAB, d_{25}-DTAB or d_{34}-DTAB at a concentration of [DTAB] = 30 mM in a $NaHCO_3/Na_2CO_3$ buffer (pD = 10.7, $I \approx 0.25$ M) in D_2O. Mono exponential fits according to the revised method of cumulants eq (3) are shown in red (h_{34}-DTAB), dark red (d_{25}-DTAB) or brown (d_{34}-DTAB). Measurement angles Θ are indicated. τ is the correlation time. Due to device-specific configurations, a maximum $g^{(2)}(\tau)$ of 0.33 is expected.

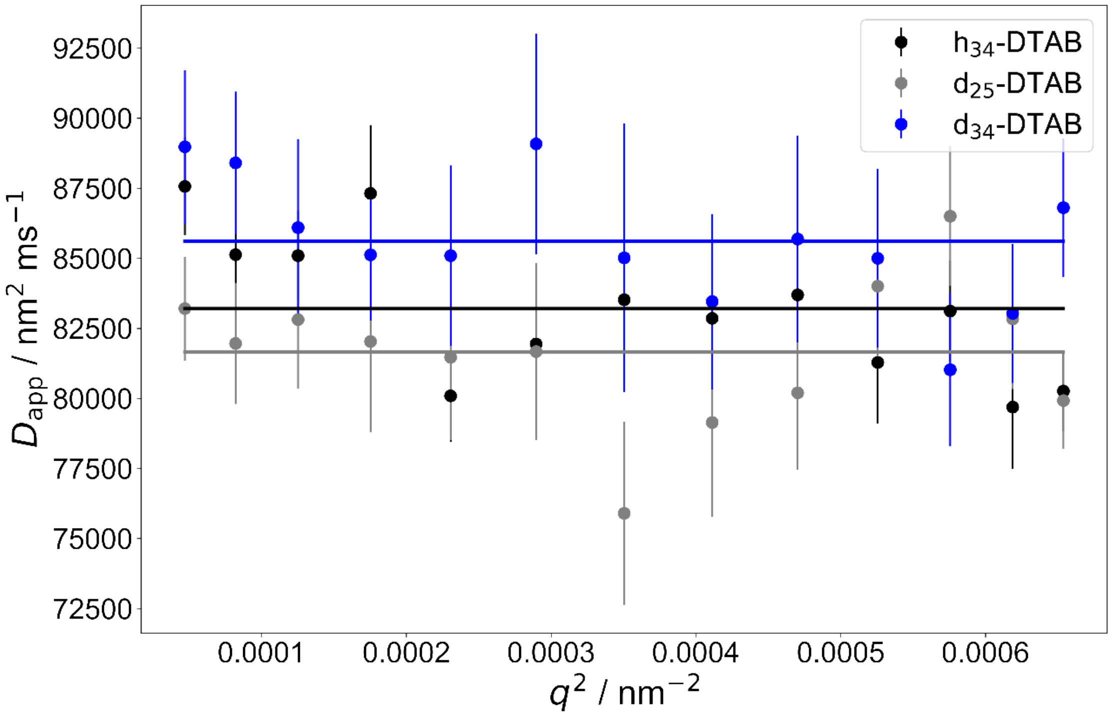

Figure SI4: Apparent diffusion coefficient obtained for DTAB micelles from mono exponential fitting of intensity correlation functions from DLS measurements at different angles according to a revised method of cumulants.[2] Correlation functions were obtained from solutions containing [DTAB] = 30 mM in a $NaHCO_3/Na_2CO_3$ buffer (pD = 10.7, $I \approx 0.25$ M) in D_2O. As no angular dependency was observed, horizontal lines indicate the average of all D_{app} for each DTAB species (black: h_{34}-DTAB, grey: d_{25}-DTAB, blue: d_{34}-DTAB).

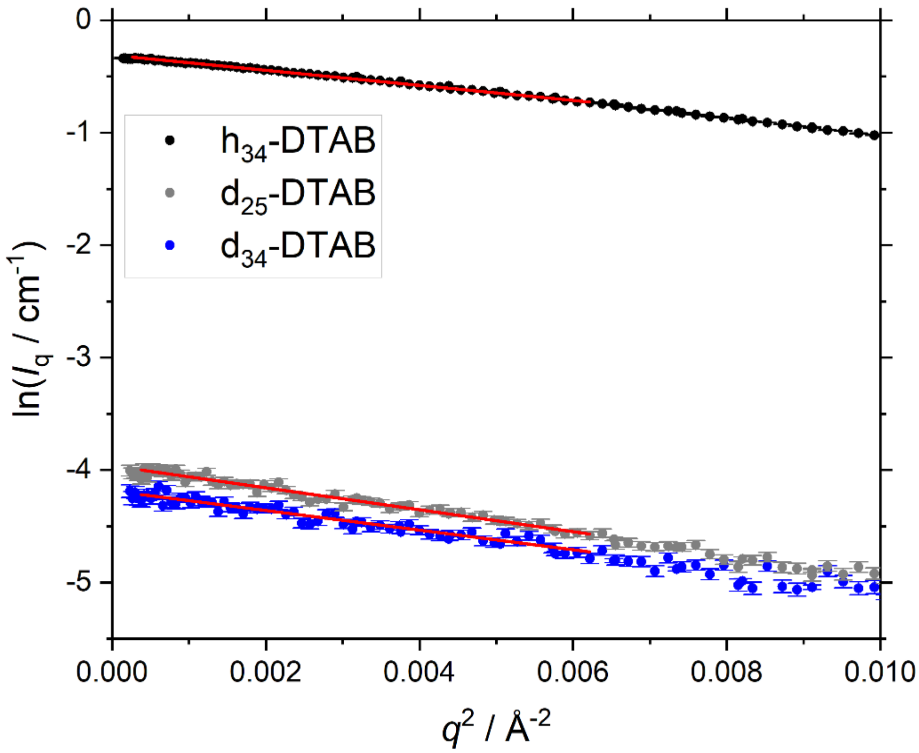

Figure SI5: Linearized Guinier plot and Guinier fits (red lines) of SANS curves of solutions containing of [DTAB] = 30 mM in a $NaHCO_3/Na_2CO_3$ buffer (pD = 10.7, $I \approx 0.25$ M) in D_2O. The color code has the same meaning as in Figure SI2.

Table SI3: Diffusion coefficient D, hydrodynamic radius R_h, radius of gyration R_g and R_g/R_h of micelles of differently deuterated DTAB species at a concentration of [DTAB] = 30 mM in a $NaHCO_3/Na_2CO_3$ buffer (pD = 10.7, $I \approx 0.25$ M) in D_2O. Diffusion coefficients were obtained by averaging apparent diffusion coefficients D_{app} obtained at different measurements angles. Their errors correspond to the standard deviation of D_{app}. Apparent diffusion coefficients are shown as a function of q in Figure SI4. Radii of gyration were obtained from a linearized Guinier analysis shown in Figure SI5.

Surfactant	D $nm^2\ ms^{-1}$	R_h Å	R_g Å	R_g/R_h
h_{34}-DTAB	8.3 ± 0.3	22.7 ± 0.8	14.18 ± 0.04	0.62 ± 0.03
d_{25}-DTAB	8.2 ± 0.3	23.1 ± 0.8	17.1 ± 0.3	0.74 ± 0.05
d_{34}-DTAB	8.6 ± 0.3	22.0 ± 0.7	16.2 ± 0.3	0.74 ± 0.05

Wormlike micelles and dependency on absolute concentration

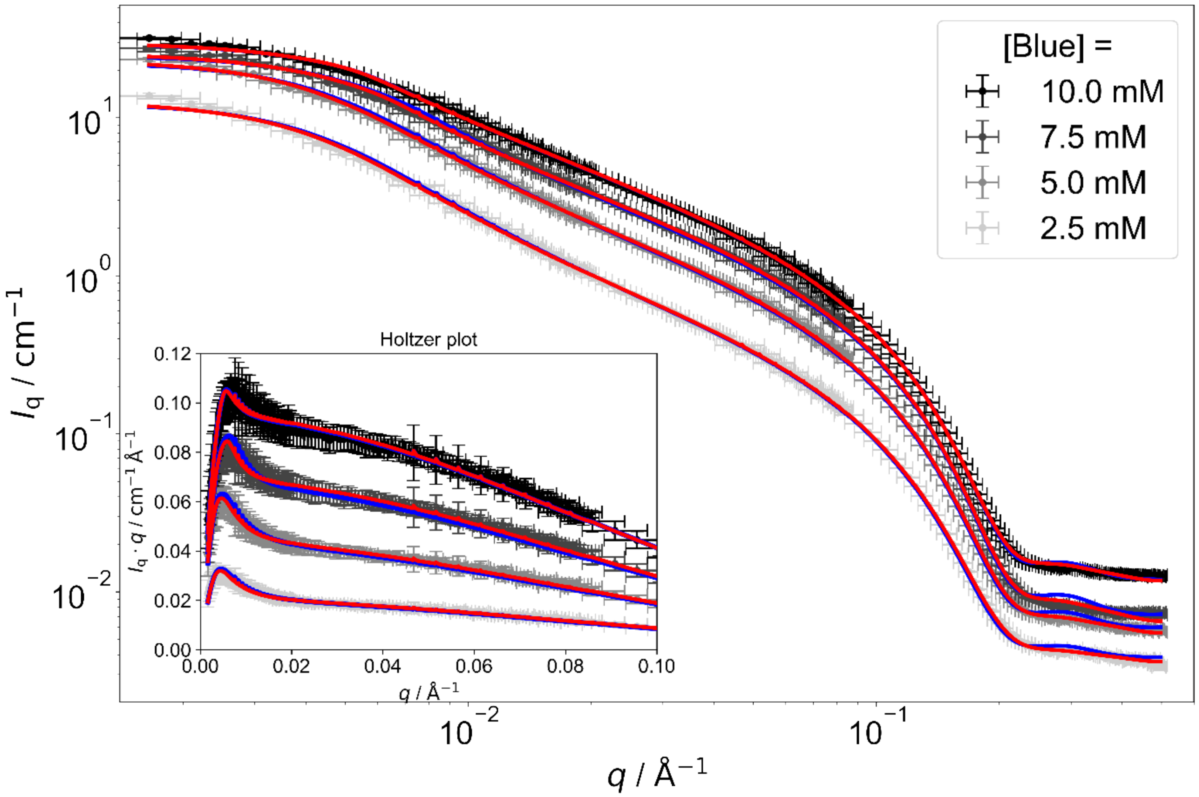

Figure SI6: Full contrast SANS curves from solutions containing Blue and DTAB at a molar ratio of [Blue]:[DTAB] = 1:3 with absolute concentrations of Blue given in the legend. Solutions were prepared in a $NaHCO_3/Na_2CO_3$ buffer (pD = 10.7, $I \approx 0.25$ M) in D_2O. The inset shows the Holtzer plot of experimental SANS curves including model fits. Red curves correspond to fits according to the form factor model of a flexible cylinder with elliptical cross section. Blue curves correspond to fits according to the form factor model of a flexible cylinder with circular cross section and set relative standard deviation of the cross section radius ($\sigma/\bar{r}$ = 0.14) considering a number-weighted Schulz distribution of the cross section radius. Fit parameters can be found in Table SI6. Reduced χ^2 values are compared in Table SI5.

Figure SI6 shows SANS curves of solutions containing Blue and DTAB with a molar ratio of [Blue]:[DTAB] = 1:3 at different absolute concentrations. The inset shows the Holtzer plot of the same curves. Three features shall be outlined in detail. (1) A significant scattering signal due to the formation of micellar assemblies is observed from a sample containing [Blue] = 2.5 mM and [DTAB] = 7.5 mM. [DTAB] = 7.5 mM lays below the critical micelle concentration (cmc) of pure DTAB (9 mM in the employed buffer system), which shows that the addition of Blue lowers the cmc of pure DTAB. This was reported in a previous publication and is commonly observed for the addition of solute to surfactant systems.[19,20] (2) All SANS curves are well described with the form factor model of a flexible cylinder with elliptical cross section with cross section dimensions r_{minor} and r_{major} remaining almost constant for all samples (Table SI4). In addition to a form factor fit according to this model, Figure SI6 also shows form factor fits of the same curves using the form factor model of a flexible cylinder with circular cross section and polydispersity in the cross section radius. This polydispersity was considered by a number-weighted Schulz distribution of the cross section radius and the relative standard deviation of the number average cross section radius was fixed to $\sigma/\bar{r}$ = 0.14. As visible from Figure SI6 at q > 0.2 Å^{-1}, SANS curves are better described with an elliptical rather than a polydisperse circular cross section. In order to well describe the SANS curves using

a circular cross section, the polydispersity of the radius would have to be increased, which is less reasonable for the presented system, as the defined hydrophobic chain length and head group size of DTAB molecules does not permit a strong variation in cross section dimensions. (3) The flexibility of wormlike micelles formed from Blue and DTAB is confirmed by a distinct maximum in the Holtzer plot of experimental SANS curves (inset in Figure SI6), which verifies that the contour length *L* of these micelles exceeds their persistence length l_p.[21] Considering the dynamic equilibrium in micellar solutions and the length polydispersity found for cylindrical Blue-DTAB micelles (Table 1, main document), a size distribution of the contour length *L* of flexible cylinders is likely.[22] However, to avoid overparameterization of the fit, this was not considered in the analysis shown in Figure SI6, leading to a systematic deviation of the employed form factor model from the data towards lower forward scattering intensities in the low-*q* region. Results from fitting the form factor of a flexible cylinder with elliptical cross section to the four experimental SANS curves are shown in Table SI4.

Table SI4: Parameters from fitting the form factor model of flexible cylinders with elliptical cross section to full contrast SANS-curves of solutions containing Blue and DTAB at a molar ratio of 1:3 in an aqueous $NaHCO_3/Na_2CO_3$ buffer with pD = 10.7 and $I \approx 0.25$ M. Corresponding fits are shown as red curves in Figure SI6 and Figure SI7.

$[Blue]$	$[DTAB]$	Cross section r_{minor} Å	r_{major} Å	$L_{contour}$ Å	l_p Å	*SLD* 10^{-6} Å^{-2}	Red. χ^2
2.5	7.5	13.79 ± 0.03	21.2 ± 2	2049 ± 25	170 ± 1	2.289 ± 0.006	2.0045
5	15	13.92 ± 0.02	21.19 ± 0.07	1705 ± 12	209 ± 1	2.113 ± 0.004	2.7727
7.5	22.5	13.99 ± 0.01	21.22 ± 0.04	1184 ± 8	257 ± 2	2.001 ± 0.003	8.4383
10	30	14.05 ± 0.01	21.15 ± 0.04	1000 ± 6	300 ± 3	1.910 ± 0.002	12.326
Average		**13.94**	**21.18**		**234**		

It can be seen, that an increase in absolute concentration results in a decrease of the contour length and absolute scattering length density of the assembly (*SLD*), whereas the persistence length l_p apparently increases. As *L* is not significantly larger than l_p, the value obtained for l_p likely contains a relatively high error and it is not clear whether the observed trend for l_p is real or an artefact from the applied fitting procedure. For this reason, a separate form factor fit was performed, where l_p was kept constant to the average of l_p for all four samples. Furthermore, the cross section dimension of the flexible elliptical cylinder was kept constant in the second fit, so that only *L* and the *SLD* of the assembly were varied. Polydispersity of the contour length needed to be introduced to describe the low-*q* region well. Resulting fits are compared to the model fits already shown in Figure SI6 in Figure SI7.

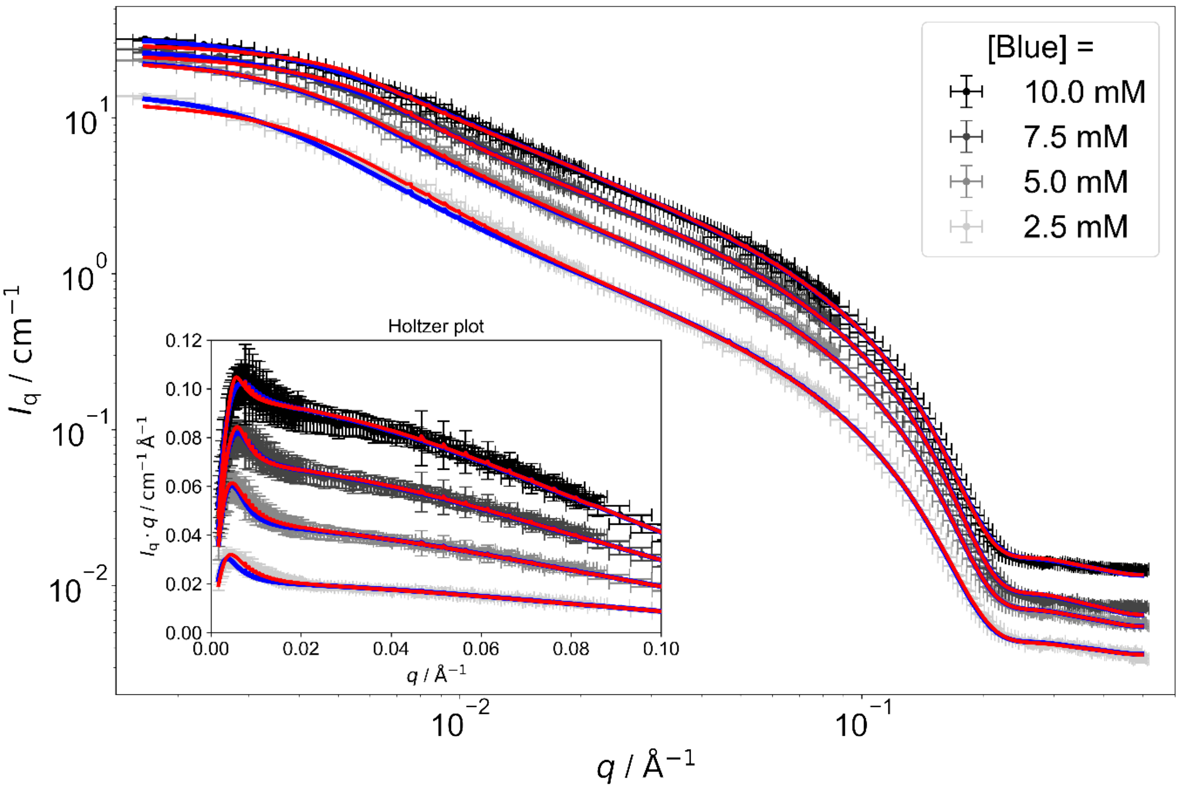

Figure SI7: Full contrast SANS-curves from solutions containing Blue and DTAB at a molar ratio of [Blue]:[DTAB] = 1:3 with absolute concentrations of Blue given in the legend. Solutions were prepared in a $NaHCO_3/Na_2CO_3$ buffer (pD = 10.7, $I \approx 0.25$ M) in D_2O. The inset shows the Holtzer plot of experimental SANS curves including model fits. Red curves correspond to fits according to the form factor model of a flexible cylinder with elliptical cross section, which was employed first. Corresponding parameters were given in Table SI4. Blue lines display form factor fits according to the same model where the persistence length l_p was kept constant and a number-weighted Schulz distribution of the contour length L was assumed. Fit parameters are visualized in Figure SI8 and reduced χ^2 values compared in Table SI5.

As anticipated, introduction of a polydispersity for L improved the fit in the low-q region. However, overall fit quality was slightly worse compared to the initial fit for two of the four SANS curves, which can be seen from Table SI5 by comparing corresponding reduced χ^2 parameters. This likely results from keeping l_p constant. Nevertheless, parameters resulting from the described analysis are displayed in Figure SI8.

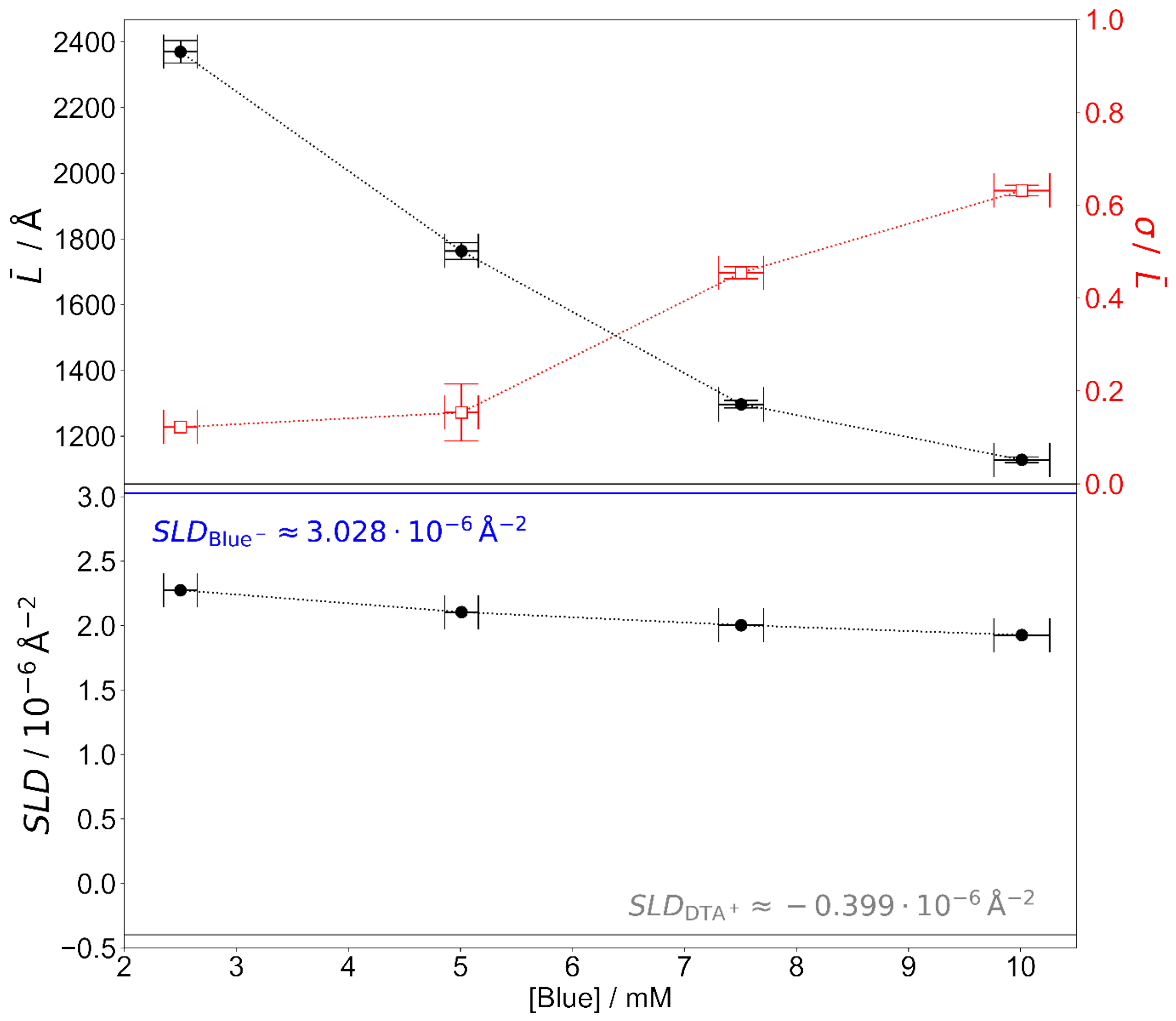

Figure SI8: Parameters resulting from fitting the form factor model of flexible cylinders with elliptical cross section to SANS curves of samples containing Blue and DTAB at a molar ratio of 1:3. The persistence length l_p = 234 Å and dimensions of the cylinder cross section (r_{minor} = 13.94 Å, r_{major} = 21.18 Å) were kept constant (Table SI4). The number average contour length $\bar{L}$, the relative standard deviation of the number-average contour length assuming a Schulz distribution ($\sigma/\bar{L}$) as well as the *SLD* of the assembly were fitted. Fits are shown as blue curves in Figure SI7. Reduced χ^2-values can be found in Table SI5.

Analogous to the initial analysis, a decrease in contour length and *SLD* is observed when increasing the absolute concentration of Blue and DTAB at constant molar ratio. The consistency of observed trends for both fitting procedures permits the following conclusive interpretation of these results. An increase in absolute concentration results in a shortening of wormlike micelles formed from Blue and DTAB, increasing the relative amount of cylinder end caps in the system and therefore the spontaneous curvature of Blue-DTAB assemblies. An increase of the spontaneous curvature of assemblies with increasing total concentration was previously reported in literature: Bergström and Pedersen observed that an increase in the absolute concentration of mixed DTAB/SDS micelles resulted in a decrease of micelle size, which was caused by an increase in spontaneous curvature of the assemblies.[23] Similarly, Verma et al. observed the increase of the spontaneous curvature of cetyltrimethylammonium-bromide/sodium salicylate bilayer structures which transformed to wormlike micelles at higher absolute

concentrations.[24] According to references 23 and 24, the increase in the spontaneous curvature of assemblies with increasing absolute concentration is easily explained by an increase of DTAB-concentration within dye-surfactant micelles upon increasing total concentration. The discrepancy between the overall DTAB content of the solution and the amount of DTAB in the Blue-surfactant co-assembly is caused by a cmc, which keeps some DTAB molecules as free monomers in bulk solution. The concentration of free monomer corresponds to the cmc of DTAB in the given system. Considering a constant concentration of free monomer this means that at higher absolute DTAB concentrations the ratio between the number of DTAB molecules participating in micelle formation and the number of monomeric DTAB molecules is higher compared to lower absolute DTAB concentrations. Therefore, higher absolute concentrations lead to a higher percentage of DTAB molecules participating in micelle formation, which leads to a decrease in the contour length. This assumption is reinforced by the observation of a decreasing *SLD* of Blue-DTAB assemblies with higher absolute concentrations, indicating that the *SLD* of the co-assembly gets closer to the *SLD* of DTAB or of the dodecyltrimethylammonium cation DTA^+, which is given in Figure SI8.

To conclude this section, the formation of wormlike micelles with elliptical cross section was observed for samples containing Blue and DTAB at a molar ratio of [Blue]:[DTAB] = 1:3. The cross section dimensions were observed to be similar for all samples. An increase in absolute concentration resulted in shortening of the wormlike micelles, which is easily explained by an increase of the [DTAB]:[Blue] ratio in the Blue-DTAB assemblies at higher absolute concentrations and a concomitant reduction in spontaneous curvature of the assembly.

Table SI5: Comparison of reduced χ^2 values for different fits to SANS curves of samples containing Blue and DTAB at a molar ratio of 1:3 in a $NaHCO_3/Na_2CO_3$ buffer (pD = 10.7, $I \approx 0.25$ M) in D_2O.

$[Blue]$	$[DTAB]$	Flexible cylinder with elliptical cross section<br>Red. χ^2	Flexible cylinder with circular cross section and $\sigma/\bar{r}$ = 0.14<br>Red. χ^2	Flexible cylinder with elliptical cross section and l_p = 234 Å<br>Red. χ^2
2.5	7.5	2.00	12.12	6.60
5	15	2.78	40.13	4.35
7.5	22.5	8.44	88.01	7.67
10	30	12.33	17.20	12.35

Table SI6: Complete parameter sets from form factor fitting of experimental SANS curves. The reduced χ^2 parameter is displayed. The scattering length density of the solvent was fixed to $SLD_{solvent}$ = 6.376 · 10^{-6} $Å^{-2}$ for all samples except for the sample containing [Blue] = 5 mM and [DTAB] = 30 mM in the full contrast measurement, where $SLD_{solvent}$ was fixed to 2.918 · 10^{-6} $Å^{-2}$ according to solvent composition. Fits assuming an elliptical assembly cross section are compared to fits assuming a circular assembly cross section with fixed relative standard deviation of the cross section radius ($\sigma/\bar{r}$ = 0.14). A number-weighted Schulz distribution of the cross section radius was assumed, hence $\bar{r}$ corresponds to the number average cross section radius. Polydispersity in the cross section radius was not considered for fitting SANS curves from samples within which the *SLD* of DTAB was matched to $SLD_{solvent}$ to avoid over parameterization. In cases where the form factor model of a cylinder was used, a number-weighted Schulz distribution of cylinder length with a relative standard deviation of $\sigma/\bar{L}$ = 0.95 was assumed with $\bar{L}$ being the number-average cylinder length. In cases, where the form factor model of a triaxial ellipsoid was used, the following relation between radii of the three semi-axes was respected: $r_{minor} < r_{major} < L$ for full contrast SANS curves and $r_{core,minor} < r_{core,major} < r_{core,L}$ for DTAB matched SANS curves. In core-shell models *th* is the shell thickness.

		Elliptical cross section						Circular cross section					
			Cross section radii						Cross section radius				
$[Blue]$<br>mM	$[DTAB]$<br>mM	**Mode**/Model	r_{minor}<br>Å	r_{major}<br>Å	Length<br>Å	SLD<br>10^{-6} $Å^{-2}$	Red.<br>χ^2	**Mode**/Model	r<br>Å	$\sigma/\bar{r}$	Length<br>Å	SLD<br>10^{-6} $Å^{-2}$	Red.<br>χ^2
5	30	**Full contrast/**<br>Triaxial ellipsoid	15.3 ± 0.2	22.0 ± 0.3	32 ± 0.3	5.778<br>± 0.004	1.319	**Full contrast/**<br>Ellipsoid	$\bar{r}$ = 17.5<br>± 0.2	$\sigma/\bar{r}$ =<br>0.14<br>(fixed)	31.8 ± 0.0.6	5.78<br>± 0.02	1.259
		**DTAB matched/**<br>Triaxial core-shell ellipsoid	$r_{core,minor}$ =<br>14.4<br>*th* = 4.7<br>± 0.9	$r_{core,major}$ =<br>20.7<br>*th* = 4.7<br>± 0.9	$r_{core,L}$ = 30<br>(fixed)	5.4<br>± 0.2	1.424	**DTAB matched/**<br>Core-shell ellipsoid	r_{core} = 19.8<br>± 0.7<br>*th* = 1.4<br>± 1.1	-	$r_{core,L}$ = 34<br>± 2<br>*th* = 1.4<br>± 1.1	5.1<br>± 0.4	1.350
		**DTAB matched/**<br>Triaxial core-shell ellipsoid	$r_{core,minor}$ =<br>14.4<br>*th* = 1.59<br>± 0.02	$r_{core,major}$ =<br>20.7<br>*th* = 1.59<br>± 0.02	$r_{core,L}$ = 30<br>(fixed)	3.028<br>(fixed)	1.563	**DTAB matched/**<br>Core-shell ellipsoid	r_{core} = 20.3<br>± 0.4<br>*th* = 0.512<br>± 0.008	-	$r_{core,L}$ = 34.5<br>± 0.7<br>*th* = 0.512<br>± 0.008	3.028<br>(fixed)	1.342
5	22.5	**Full contrast/**<br>Elliptical cylinder	14.78<br>± 0.02	21.21<br>± 0.06	$\bar{L}$ = 73.4 ± 2<br>$\sigma/\bar{L}$ = 0.95	1.135<br>± 0.001	16.20	**Full contrast/**<br>Cylinder	$\bar{r}$ = 16.891<br>± 0.005	$\sigma/\bar{r}$ =<br>0.14<br>(fixed)	$\bar{L}$ = 74.5<br>± 0.2<br>$\sigma/\bar{L}$ = 0.95	1.130<br>± 0.001	18.62
		**DTAB matched/**<br>Elliptical core-shell cylinder	$r_{core,minor}$<br>=13.4 ± 0.4<br>*th* = 3.3<br>± 0.5	$r_{core,major}$ =<br>19.2 ± 0.5<br>*th* = 3.3<br>± 0.5	$\bar{L}$ = 73<br>$\sigma/\bar{L}$ = 0.95<br>(fixed)	5.6<br>± 0.2	1.963	**DTAB matched/**<br>Core-shell cylinder	r_{core} = 17.1<br>± 0.6<br>*th* = 3.6<br>± 0.7	-	$\bar{L}$ = 74.5<br>$\sigma/\bar{L}$ = 0.95<br>(fixed)	5.34<br>± 0.08	4.468
		**DTAB matched/**<br>Elliptical core-shell cylinder	$r_{core,minor}$ =<br>14.7 ± 0.3<br>*th* = 0.748<br>± 0.006	$r_{core,major}$ =<br>21.2 ± 0.4<br>*th* = 0.748<br>± 0.006	$\bar{L}$ = 73<br>$\sigma/\bar{L}$ = 0.95<br>(fixed)	3.028<br>(fixed)	1.768	**DTAB matched/**<br>Core-shell cylinder	r_{core} = 17.5<br>± 0.3<br>*th* = 0.333<br>± 0.005	-	$\bar{L}$ = 74.5<br>$\sigma/\bar{L}$ = 0.95<br>(fixed)	3.028<br>(fixed)	1.627

7.5	30	**Full contrast/** Elliptical cylinder	14.336 ± 0.009	21.19 ± 0.04	$\bar{L}$ = 148 ± 1 σ/$\bar{L}$ = 0.95	1.059 ± 0.001	15.71	**Full contrast/** Cylinder	$\bar{r}$ = 16.621 ± 0.003	σ/$\bar{r}$ = 0.14 (fixed)	$\bar{L}$ = 151.0 ± 0.4 σ/$\bar{L}$ = 0.95	1.061 ± 0.001	20.43
		**DTAB matched/** Elliptical core-shell cylinder	$r_{core,minor}$ = 14.5 ± 0.2 *th* = 2.1 ± 0.4	$r_{core,major}$ = 21.4 ± 0.3 *th* = 2.1 ± 0.4	$\bar{L}$ = 148 σ/$\bar{L}$ = 0.95 (fixed)	5.0 ± 0.2	2.717	**DTAB matched/** core-shell cylinder	r_{core} = 16.9 ± 0.3 *th* = 4.0 ± 0.4	-	$\bar{L}$ = 151 σ/$\bar{L}$ = 0.95 (fixed)	5.34 ± 0.06	8.276
		**DTAB matched/** Elliptical core-shell cylinder	$r_{core,minor}$ = 15.7 ± 0.2 *th* = 0.874 ± 0.004	$r_{core,major}$ = 23.3 ± 0.2 *th* = 0.874 ± 0.004	$\bar{L}$ = 148 σ/$\bar{L}$ = 0.95 (fixed)	3.028 (fixed)	8.189	**DTAB matched/** core-shell cylinder	r_{core} = 18.6 ± 0.2 *th* = 0.573 ± 0.004	-	$\bar{L}$ = 151 σ/$\bar{L}$ = 0.95 (fixed)	3.028 (fixed)	8.245
2.5	7.5	**Full contrast/** Elliptical flexible cylinder	13.79 ± 0.03	21.2 ± 2	*L* = 2049 ± 25 l_p = 170 ± 1	2.289 ± 0.006	2.005	**Full contrast/** Flexible cylinder	$\bar{r}$ = 17.108 ± 0.009	σ/$\bar{r}$ = 0.14 (fixed)	*L* = 2057 ± 22 l_p = 153 ± 2	1.406 ± 0.003	12.12
		**DTAB matched/** Elliptical core-shell cylinder	$r_{core,minor}$ = 10.3 ± 0.6 *th* = 4.1 ± 1.0	$r_{core,major}$ = 15.6 ± 0.9 *th* = 4.1 ± 1.0	$L = l_p$ = 170 (fixed)	5.1 ± 0.4	2.009	**DTAB matched/** Core-shell cylinder	r_{core} = 11.6 ± 1.2 *th* = 4.7 ± 0.5	-	$L = l_p$ = 153 (fixed)	4.8 ± 0.2	1.717
		**DTAB matched/** Elliptical core-shell cylinder	$r_{core,minor}$ = 11.5 ± 0.4 *th* = 1.43 ± 0.03	$r_{core,major}$ = 17.4 ± 0.5 *th* = 1.43 ± 0.03	$L = l_p$ = 170 (fixed)	3.028 (fixed)	1.708	**DTAB matched/** Core-shell cylinder	r_{core} = 14.0 ± 0.4 *th* = 1.03 ± 0.03	-	$L = l_p$ = 153 (fixed)	3.028 (fixed)	1.612
5	15	**Full contrast/** Elliptical flexible cylinder	13.92 ± 0.02	21.19 ± 0.07	*L* = 1705 ± 12 l_p = 209 ± 1	2.113 ± 0.004	2.773	**Full contrast/** Flexible cylinder	$\bar{r}$ = 17.091 ± 0.006	σ/$\bar{r}$ = 0.14 (fixed)	*L* = 1714 ± 14 l_p = 178 ± 2	1.199 ± 0.002	40.13
		**DTAB matched/** Elliptical core-shell cylinder	$r_{core,minor}$ = 10.9 ± 0.5 *th* = 3.5 ± 0.8	$r_{core,major}$ = 16.5 ± 0.7 *th* = 3.5 ± 0.8	$L = l_p$ = 209 (fixed)	4.9 ± 0.3	1.143	**DTAB matched/** Core-shell cylinder	r_{core} = 12.6 ± 0.8 *th* = 4.9 ± 0.4	-	$L = l_p$ = 178 (fixed)	4.93 ± 0.07	2.182
		**DTAB matched/** Elliptical core-shell cylinder	$r_{core,minor}$ = 11.7 ± 0.3 *th* = 1.41 ± 0.02	$r_{core,major}$ = 17.8 ± 0.4 *th* = 1.41 ± 0.02	$L = l_p$ = 209 (fixed)	3.028 (fixed)	2.066	**DTAB matched/** Core-shell cylinder	r_{core} = 14.6 ± 0.3 *th* = 0.97 ± 0.02	-	$L = l_p$ = 178 (fixed)	3.028 (fixed)	1.947

		**Full contrast/** Elliptical flexible cylinder	13.99 ± 0.01	21.22 ± 0.04	L = 1184 ± 8 l_p = 257 ± 2	2.001 ± 0.003	8.438	**Full contrast/** Flexible cylinder	$\bar{r}$ = 17.074 ± 0.004	$\sigma/\bar{r}$ = 0.14 (fixed)	L = 1200 ± 7 l_p = 214 ± 2	1.067 ± 0.002	88.01
7.5	22.5	**DTAB matched/** Elliptical core-shell cylinder	$r_{core,minor}$ = 11.1 ± 0.5 th = 4.0 ± 0.8	$r_{core,major}$ = 16.9 ± 0.7 th = 4.0 ± 0.8	$L = l_p$ = 257 (fixed)	5.3 ± 0.3	2.008	**DTAB matched/** Core-shell cylinder	r_{core} = 13.3 ± 0.7 th = 5.0 ± 0.5	-	$L = l_p$ = 214 (fixed)	5.10 ± 0.06	1.311
		**DTAB matched/** Elliptical core-shell cylinder	$r_{core,minor}$ = 12.3 ± 0.3 th = 1.22 ± 0.02	$r_{core,major}$ = 18.7 ± 0.4 th = 1.22 ± 0.02	$L = l_p$ = 257 (fixed)	3.028 (fixed)	1.139	**DTAB matched/** Core-shell cylinder	r_{core} = 15.6 ± 0.3 th = 0.70 ± 0.02	-	$L = l_p$ = 214 (fixed)	3.028 (fixed)	1.130
		**Full contrast/** Elliptical flexible cylinder	14.05 ± 0.01	21.15 ± 0.04	L = 1000 ± 6 l_p = 300 ± 3	1.910 ± 0.002	12.33	**Full contrast/** Flexible cylinder	$\bar{r}$ = 17.039 ± 0.004	$\sigma/\bar{r}$ = 0.14 (fixed)	L = 1002 ± 6 l_p = 293 ± 3	0.899 ± 0.002	17.20
10	30	**DTAB matched/** Elliptical core-shell cylinder	$r_{core,minor}$ = 12.0 ± 0.8 th = 2.4 ± 1.2	$r_{core,major}$ = 18.3 ± 1.2 th = 2.4 ± 1.2	$L = l_p$ = 300 (fixed)	4.5 ± 0.3	1.691	**DTAB matched/** Core-shell cylinder	r_{core} = 13.7 ± 0.5 th = 4.7 ± 0.4	-	$L = l_p$ = 293 (fixed)	5.02 ± 0.05	1.926
		**DTAB matched/** Elliptical core-shell cylinder	$r_{core,minor}$ = 12.6 ± 0.2 th = 1.28 ± 0.02	$r_{core,major}$ = 19.1 ± 0.2 th = 1.28 ± 0.02	$L = l_p$ = 300 (fixed)	3.028 (fixed)	2.000	**DTAB matched/** Core-shell cylinder	r_{core} = 15.6 ± 0.2 th = 0.761 ± 0.006	-	$L = l_p$ = 293 (fixed)	3.028 (fixed)	1.967

NOESY Spectrum

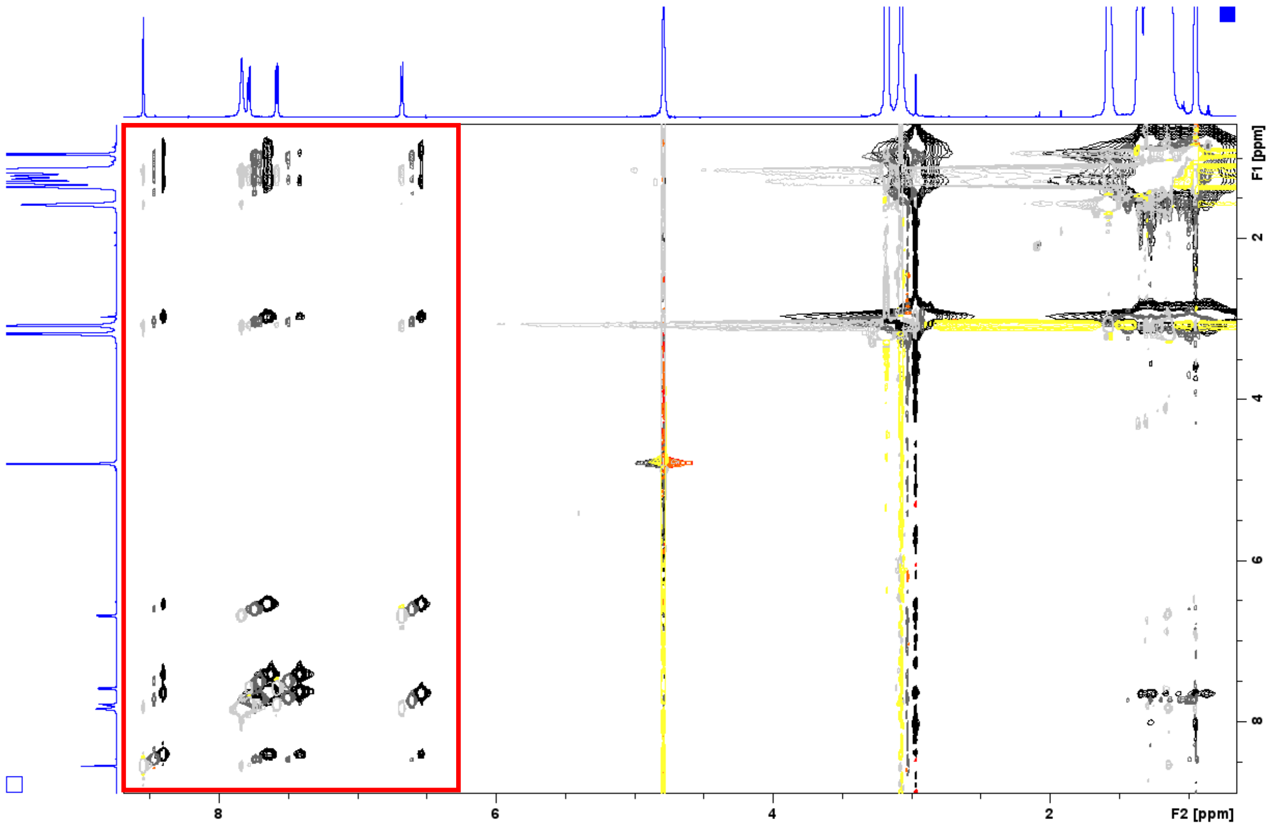

Figure SI9: NOESY spectra of solutions containing [DTAB] = 30 mM and varying concentrations of Blue. The solvent is a $NaHCO_3/Na_2CO_3$ buffer (pD = 10.7, $I \approx 0.25$ M) prepared in D_2O. Negative peaks are displayed in light grey, dark grey and black for samples containing [Blue] = 5 mM, 7.5 mM and 10 mM respectively. Positive peaks are displayed in yellow, orange and red for samples containing [Blue] = 5 mM, 7.5 mM and 10 mM respectively. The red square marks the section shown in Figure 11 in the main document. The ^{1}H-NMR spectra of the sample containing [Blue] = 5 mM and [DTAB] = 30 mM are displayed on the sides. Addition of Blue to a solution with constant DTAB concentration causes an upfield shift of some DTAB resonances, going along with peak broadening. Furthermore, an increase of the [Blue]:[DTAB] molar ratio causes an upfield shift of all Blue resonances. Therefore, peak positions in the NOESY spectrum depend on [Blue].

References

(1) Garland, C. W.; Nibler, J. W.; Shoemaker, D. P. *Experiments in Physical Chemistry*, 8th ed.; McGraw-Hill Higher Education: Boston, 2009.

(2) Frisken, B. J. Revisiting the Method of Cumulants for the Analysis of Dynamic Light-Scattering Data. *Appl. Opt.* **2001**, *40* (24), 4087–4091. https://doi.org/10.1364/AO.40.004087.

(3) Schärtl, W. *Light Scattering from Polymer Solutions and Nanoparticle Dispersions*; Springer Laboratory; Springer Berlin Heidelberg: Berlin, Heidelberg, 2007. https://doi.org/10.1007/978-3-540-71951-9.

(4) *Neutrons, X-Rays, and Light: Scattering Methods Applied to Soft Condensed Matter*, 1st ed.; Lindner, P., Zemb, T., Eds.; North-Holland delta series; Elsevier: Amsterdam ; Boston, 2002.

(5) Berr, S. S. Solvent Isotope Effects on Alkytrimethylammonium Bromide Micelles as a Function of Alkyl Chain Length. *J. Phys. Chem.* **1987**, *91* (18), 4760–4765. https://doi.org/10.1021/j100302a024.

(6) Hayter, J. B.; Penfold, J. Determination of Micelle Structure and Charge by Neutron Small-Angle Scattering. *Colloid & Polymer Sci* **1983**, *261* (12), 1022–1030. https://doi.org/10.1007/BF01421709.

(7) Marcus, Y. *Ion Properties*; Marcel Dekker: New York, 1997.
(8) Lide, D. R. *CRC Handbook of Chemistry and Physics*; CRC Press: Boca Raton, FL, 2005.
(9) Conway, B. E. The Evaluation and Use of Properties of Individual Ions in Solution. *J Solution Chem* **1978**, *7* (10), 721–770. https://doi.org/10.1007/BF00643580.
(10) Hayter, J. B.; Penfold, J. An Analytic Structure Factor for Macroion Solutions. *Molecular Physics* **1981**, *42* (1), 109–118. https://doi.org/10.1080/00268978100100091.
(11) Hansen, J.-P.; Hayter, J. B. A Rescaled MSA Structure Factor for Dilute Charged Colloidal Dispersions. *Molecular Physics* **1982**, *46* (3), 651–656. https://doi.org/10.1080/00268978200101471.
(12) Vidulich, G. A.; Evans, D. F.; Kay, R. L. The Dielectric Constant of Water and Heavy Water between 0 and 40 Degree. *J. Phys. Chem.* **1967**, *71* (3), 656–662. https://doi.org/10.1021/j100862a028.
(13) Feigin, L. A.; Svergun, D. I. *Structure Analysis by Small-Angle X-Ray and Neutron Scattering*; Springer US, 1987. https://doi.org/10.1007/978-1-4757-6624-0.
(14) Kotlarchyk, M.; Chen, S. Analysis of Small Angle Neutron Scattering Spectra from Polydisperse Interacting Colloids. *J. Chem. Phys.* **1983**, *79* (5), 2461–2469. https://doi.org/10.1063/1.446055.
(15) Bergström, M.; Pedersen, J. Structure of Pure SDS and DTAB Micelles in Brine Determined by Small-Angle Neutron Scattering (SANS). *PHYS CHEM CHEM PHYS* **1999**, *1*, 4437–4446. https://doi.org/10.1039/a903469b.
(16) Berr, S. S.; Caponetti, E.; Johnson, J. S. Jr.; Jones, R. R. M.; Magid, L. J. Small-Angle Neutron Scattering from Hexadecyltrimethylammonium Bromide Micelles in Aqueous Solutions. *J. Phys. Chem.* **1986**, *90* (22), 5766–5770. https://doi.org/10.1021/j100280a059.
(17) Zana, R.; Picot, C.; Duplessix, R. Effect of Alcohol on the Properties of Micellar Systems. V. Small Angle Neutron Scattering Study. *Journal of Colloid and Interface Science* **1983**, *93* (1), 43–53. https://doi.org/10.1016/0021-9797(83)90382-X.
(18) Tabony, J. Structure of the Polar Head Layer and Water Penetration in a Cationic Micelle. *Molecular Physics* **1984**, *51* (4), 975–989. https://doi.org/10.1080/00268978400100641.
(19) Müller, W.; Schweins, R.; Nöcker, B.; Kohlbrecher, Joachim; Smales, G. J.; Huber, K. Comparative Study of the Co-Assembly Behaviour of 3-Chloro-4 Hydroxy-Phenylazo Dyes with DTAB. *Soft Matter* **2023**, *19* (24), 4588–4598. https://doi.org/10.1039/D3SM00501A.
(20) Rosen, M. J. *Surfactants and Interfacial Phenomena*, 3rd ed.; Wiley-Interscience; John Wiley & Sons, Inc: Hoboken, New Jersey, USA, 2004.
(21) A. Dreiss, C. Wormlike Micelles: Where Do We Stand? Recent Developments, Linear Rheology and Scattering Techniques. *Soft Matter* **2007**, *3* (8), 956–970. https://doi.org/10.1039/B705775J.
(22) Patist, A.; Oh, S. G.; Leung, R.; Shah, D. O. Kinetics of Micellization: Its Significance to Technological Processes. *Colloids and Surfaces A: Physicochemical and Engineering Aspects* **2001**, *176* (1), 3–16. https://doi.org/10.1016/S0927-7757(00)00610-5.
(23) Bergström, M.; Pedersen, J. S. Formation of Tablet-Shaped and Ribbonlike Micelles in Mixtures of an Anionic and a Cationic Surfactant. *Langmuir* **1999**, *15* (7), 2250–2253. https://doi.org/10.1021/la981495x.
(24) Verma, G.; Kumar, S.; Schweins, R.; K. Aswal, V.; A. Hassan, P. Transition from Long Micelles to Flat Bilayers Driven by Release of Hydrotropes in Mixed Micelles. *Soft Matter* **2013**, *9* (17), 4544–4552. https://doi.org/10.1039/C3SM27913H.
